# Loss of NLN suppresses lung cancer progression by inducing ferroptosis through downregulating m^6^A methylation of GPX4

**DOI:** 10.1016/j.redox.2025.103928

**Published:** 2025-11-11

**Authors:** Lei Liu, Yinyun Ni, Ying Yang, Gan Zhang, Shengqiang Mao, Ningning Chao, Menglin Yao, Chengdi Wang, Li Zhang

**Affiliations:** a Department of Pulmonary and Critical Care Medicine, Institute of Respiratory Health, State Key Laboratory of Respiratory Health and Multimorbidity, Frontiers Science Center for Disease-related Molecular Network, Sichuan Provincial Engineering Laboratory of Precision Medicine, Precision Medicine Key Laboratory of Sichuan Province, West China Hospital, West China School of Medicine, Sichuan University, Chengdu, Sichuan Province, 610041, China; b Laboratory of Metabolomics and Gynecological Disease Research, Key Laboratory of Birth Defects and Related Diseases of Women and Children of Ministry of Education, West China Second University Hospital, Sichuan University, Chengdu, Sichuan, China

**Keywords:** Non-small cell lung cancer, Neurolysin (NLN), Ferroptosis, m^6^A modification

## Abstract

Current targeted therapies for non-small cell lung cancer (NSCLC) face significant limitations, primarily due to the small proportion of patients who respond to existing targets and the development of drug resistance. Consequently, identifying novel therapeutic targets is crucial to overcome these challenges. In this study, we identified Neurolysin (NLN) as a novel therapeutic target for NSCLC using high-throughput proteomics. We validated the oncogenic role of NLN through in vitro cellular models and in vivo studies, and uncovered a previously unrecognized mechanism in ferroptosis regulation. We demonstrated that NLN expression was significantly upregulated in lung cancer tissues relative to adjacent normal tissues. More notably, inhibition of NLN induced ferroptosis in lung cancer cells. In vivo studies confirmed that the suppression of NLN significantly inhibited tumor growth in a mouse model. Mechanistic investigations revealed that NLN inhibition reduced the m^6^A modification of *GPX4* mRNA, resulting in its degradation and the subsequent induction of ferroptosis. Moreover, we developed a specific small molecule inhibitor, NR2, which targets NLN and induces tumor cell death both in vitro and in vivo, showcasing potent anti-tumor activity. These findings not only establish NLN as a crucial regulator of ferroptosis in NSCLC but also provide a novel therapeutic strategy to target ferroptosis in lung cancer. Our work offers a scientific foundation for the clinical development of NLN-targeted therapies in NSCLC, addressing both the challenges of drug resistance and tumor progression.

## 1 Introduction

Lung cancer, particularly non-small cell lung cancer (NSCLC), constitutes approximately 85 % of all lung cancer cases and is the is the leading cause of cancer-related mortality globally [1,2]. Despite considerable advancements in targeted therapies and immunotherapy, the five-year survival rate for advanced-stage NSCLC remains unacceptably low, underscoring the urgent need for innovative therapeutic strategies [3,4]. The heterogeneity of lung cancer, coupled with its intricate molecular landscape, necessitates the investigation of novel molecular targets that could yield more effective treatment options.

Ferroptosis, a recently characterized form of regulated cell death that is distinct from apoptosis, necrosis, and autophagy, is induced by iron-dependent lipid peroxidation [5]. The discovery of ferroptosis has opened new avenues in cancer research, particularly due to its potential role in tumor suppression [6]. This process is primarily regulated by intracellular antioxidant systems, including glutathione peroxidase 4 (GPX4), which reduces lipid hydroperoxides, thereby preventing lipid peroxidation and ferroptosis [7]. GPX4 has emerged as a critical regulator of ferroptosis, with studies demonstrating that its inhibition leads to the accumulation of lipid peroxides, culminating in ferroptotic cell death [8]. Therefore, modulating key proteins such as GPX4 to regulate ferroptosis represents a promising strategy for cancer therapy.

Neurolysin (NLN), a mitochondrial metallopeptidase, has traditionally been recognized for its role in the degradation of neuropeptides and the regulation of mitochondrial function [9,10]. Recent studies have begun to elucidate its involvement in cancer, particularly in hematological malignancies such as acute myeloid leukemia (AML), where NLN has been demonstrated to support tumor viability by regulating the formation of mitochondrial respiratory chain supercomplexes [10]. However, the role of NLN in solid tumors, including NSCLC, remains largely unexplored, highlighting a significant gap in our understanding of its potential oncogenic functions.

Through mining public cancer databases, it is found that NLN may play a crucial role in the progression of lung cancer, potentially by modulating key processes such as cell proliferation, migration, and resistance to cell death. Mitochondria, as the main site of ROS production and iron metabolism, play a central role in the induction of ferroptosis [11]. Given its mitochondrial localization and known functions in regulating cellular metabolism, NLN could be implicated in the modulation of ferroptosis, a hypothesis that has yet to be explored in the context of lung cancer.

In this study, we investigate the role of NLN in lung cancer, specifically focusing on its potential involvement in the regulation of ferroptosis. Mechanically, we found that NLN contribute to lung cancer progression by modulating the m^6^A modification of GPX4 mRNA, which in turn inhibits ferroptosis and promotes tumor survival. By elucidating the mechanisms through which NLN influences ferroptosis, this study aims to provide new insights into the metabolic vulnerabilities of lung cancer and identify NLN as a potential therapeutic target. Our findings could have significant implications for the development of novel therapies aimed at inducing ferroptosis in NSCLC.

## 2 Methods and materials

### 2.1 Cell lines and cultures

The human lung cancer A549 cells and SK-MES-1 cells were purchased from the Chinese Academy of Sciences Cell Bank (Shanghai, China). The human MRC-5, BEAS-2B, H1299, H1975, PC-9, HCC95, H266, HEK293T, Calu-1 cells came from Core Facilities of West China Hospital (Chengdu, China). BEAS-2B, A549, H1299, H1975, PC-9, HCC95, H266, Calu-1 cells were maintained in Gibco™ BASIC RPMI 1640 Medium with 10 % fetal bovine serum (FBS). MRC-5, SK-MES-1 cells were maintained in Gibco™ MEM with 10 % FBS. HEK293T cells were maintained in Dulbecco's Modified Eagle Medium (Gibco™ DMEM) with 10 % FBS. A549-Tet-shNLN cells were maintained in RPMI 1640 medium supplemented with 10 % tetracycline-free fetal bovine serum. SK-MES-1-Tet-shNLN cells were cultured in MEM medium containing 10 % tetracycline-free fetal bovine serum, glutamine, non-essential amino acids, and sodium pyruvate. All cell lines were incubated at 37 °C in a humidified atmosphere with 5 % CO_2_.

### 2.2 Reagents

RSL3 (#S8155), Liproxstatin-1(#S7699), Necrostain-1(S8037), Z-VAD-FMK (S7023) Chloroquine(S6999) and FB23-2(#S8837) were obtained from Selleck Chemicals (Houston, TX, USA). Actinomycin D(M4881) was from AbMole (USA). Methylene Blue Solution (#G1301) was purchased from Solarbio (China). D-luciferin (ab143655) was from abcam (UK).

### 2.3 Tissue specimens

Primary LUAD (lung adenocarcinoma) and LUSC (lung squamous cell carcinoma) tissues and the corresponding non-cancerous adjacent tissues were obtained from the biobank of West China Hospital, Sichuan University.

### 2.4 Immunoblotting, immunohistochemistry and immunofluorescence

Western blot analysis was performed as followed describe. In brief, cells were extracted with cell/tissue total protein extraction kit (Abbkine). Protein concentration was determined using a BCA Protein Assay Kit (KeyGEN BioTECH). The proteins were resolved by SDS-PAGE, electrophoretically transferred to PVDF membranes. The membrane was blocked with skimmed milk for 1 h and incubated with primary antibodies (Table S1) overnight at 4 °C. For detection, membranes were incubated with Goat anti-Rabbit IgG secondary antibodies (Invitrogen) and Goat anti-Mouse IgG (Invitrogen) dissolved in antibody dilution buffer (Beyotime) for 1 h at room temperature. The membranes were visualized with chemiluminescence (Bio-Rad; Hercules, CA, USA) according to the manufacturer's protocol.

Immunohistochemistry was performed as followed describe. In brief, Tissue samples were fixed in 4 % paraformaldehyde in phosphate buffered saline (PBS) and embedded in paraffin. Sections (4 μm) were de-waxed, rehydrated, and incubated in 0.01 M citrate buffer for 20 min at 95 °C for antigen retrieval. Endogenous peroxidase activity was blocked with 3 % hydrogen peroxide and non-specific antigens were blocked with 10 % normal goat serum (Solarbio, Beijing) and stained with hematoxylin and eosin (H&E) or followed by incubation with primary antibody (Table S1) at 4 °C for 12 h. Images were scanned by VS200 digital slide scanner (Olympus).

For immunofluorescence, cells grown on poly-lysine-treated coverslides were fixed with 4 % paraformaldehyde and permeabilized with 0.1 % Triton X-100/PBS before incubated with primary antibodies and appropriate secondary antibodies: Alexa Fluor 647 goat anti-mouse IgG or Alexa Fluor 488 goat anti-Rabbit IgG from Invitrogen. Nuclei were counterstained with DAPI. Images were acquired by Laser Scanning Confocal Microscopy (Nikon Ni-E A1R MP+).

### 2.5 Mitochondrial morphology

For ultrastructural analysis of mitochondria, TEM was used. Digest and centrifuge si-NLN A549 cells and collect them in 1.5 ml EP tubes. Add 500 μl of 2.5 % Gluta fixative (Solarbio) to each tube and fix at 4 °C. Then the cell was postfixed in 1 % osmium tetroxide, dehydrated in series acetone, infiltrated in Epox 812 for a longer, and embedded. The semithin sections were stained with methylene blue and Ultrathin sections were cut with diamond knife, stained with uranyl acetate and lead citrate. Sections were examined with JEM-1400-FLASH Transmission Electron Microscope. The image acquisition was carried out by Lilai biomedicine (Chengdu, China).

To observe mitochondrial morphological changes, MitoTracker staining was described as below. A549 cells in the NC group and si-NLN group were further cultured in a 96-well blackboard for 24–48 h. Prepare Mito Tracker staining solution in serum-free medium at a ratio of 1:800 and stain for 10–15 min. Wash twice with PBS and use a High-content analysis system (PerkinElmer) for imaging.

### 2.6 CCK8 assay

CCK8 assays were performed according to the manufacturer's manual (Abmole). Briefly, 1600–2000 cells in 100 μl culture medium were added into each well of a 96-well plate for period of time. At each time point, 10 μl of sterile CCK-8 was added to each well and incubated for another 1 h at 37 °C. The absorbance at 450 nm was determined using a microplate reader.

### 2.7 Cell migration&invasion and colony-formation assay

Using 8.0 mm pore inserts (Millipore), we used sterile forceps to remove the chamber from the operating table and place it in the standard hole of a 24-well plate, forming an upper chamber and a lower chamber. Cell invasion assays were performed in 24-well plates with 8.0 mm pore inserts pre-coated with Matrigel (Corning). A549 cells were digested and resuspended in FBS-free 1640 culture medium. A total of 200 μl of A549 cells was seeded into the upper chamber, while a 600 μl complete culture medium (1640 with 10 % FBS) was added to the lower chamber. For each insert, the invading cells in five random fields were counted. After cultivation, the cells were fixed with methanol or 4 % paraformaldehyde for 15–20 min, and stained with 0.01 % crystal violet(Solarbio) staining solution for 30 min.

A549 shNLN and SK-MES-1-shNLN cells which were knocked down NLN, were seeded in 6-well plates, with 600–800 cells per well, and cultured for 10–14 days. After cultivation, the cells were fixed with methanol or 4 % paraformaldehyde for 30 min, and stained with 0.01 % crystal violet staining solution for 20–30 min, and take photos for observation.

### 2.8 Fluorescence probe

Cultivate treated cells with appropriate density and optional add BODIPY™ 581/591C11 (#D3681, ThermoFisher) for lipid peroxidation detection (working concentration: 5 μM), Liperfluo (Dojindo laboratories) for lipid peroxidation detection (working concentration:10 μmol/L), FerroOrange (Dojindo laboratories) for Fe^2+^ detection (1:1000) or Cell ROS Deep Red (Thermo Scientific) for reactive oxygen species detection. Incubate at 37 °C for 30 min and then use a flow cytometer or fluorescence microscope for detection.

### 2.9 Quantitative reduction of total glutathione (GSH) and detection of cysteine uptake

The GSH quantification and cysteine uptake detection involved in this study were performed using the GSSG/GSH Quantification Kit and Cystine Uptake Assay Kit from Dojindo Laboratories, following the instructions.

### 2.10 qRT-PCR(Real-time PCR)

RT-PCR RNA was extracted using Tissue/Cell RNA Rapid Extraction Kit (Aidlab). cDNAs were synthesized from 1 μg of total RNA using the iScript cDNA Synthesis kit (Bio-Rad). The expression of genes was measured by qRT-PCR with iTaq SYBR Green Supermix (Bio-Rad). The qRT-PCR primers used in this study are listed in Table S2.

### 2.11 Small interfering RNAs (siRNAs), plasmid and lentivirus construction, and transfection protocol

Cells were seeded at a density of 3 × 10^5^ cells/well in 6-well plates. The siRNA duplexes were transfected into cells when the cells were 70–80 % confluent. The following siRNA sequences were used to target the RNAs indicated: si-NLN-:CCAGCUACCUUUGGACAUUTT; si-NLN-2:GGUGAGGACUUACUUUCAUTT; si-NLN-3:GCUGACUUCGUCCUUGAAATT. qRT-PCR and Western blotting was used to evaluate siRNA knockdown efficiency.

The plasmids used in this study were all synthesized by YouBio biotechnology. After obtaining the plasmid, the QIAGE N EndoFree Plasma Maxi Kit (QIAGEN) with endotoxin removal was used for plasmid extraction. HEK293T cells were transfected with plasmids using Lipofectamine 3000 (Thermo Scientific) according to the manufacturer's instructions. Stable strains were constructed using lentivirus packaging and infection. After adding optim-MEM, plasmid, and lipo8000 (Beyotime Biotechnology), puromycin was used for screening A549, SK-MES-1 and H1975 cells overexpressing NLN and those with NLN knockdown, such as A549-shNLN, H1975-NLN cell lines. For NLN knockdown induction, cells were treated with 5 μg/mL doxycycline for 72 h to achieve consistent gene silencing efficiency across experiments and designated as A549-Tet-shNLN and SK-MES-1-Tet-shNLN.

### 2.12 RNA dot blot

To perform an RNA Dot Blot analysis, briefly, start by diluting the RNA sample in a gradient and heating it to disrupt secondary structures, then quickly cool it on ice. Apply the samples to a nitrocellulose membrane, dry, and crosslink the RNA. Wash the membrane, block it, and incubate with a primary antibody specific to m^6^A at 4 °C overnight. After washing, apply a HRP-conjugated secondary antibody and incubate. Wash again, develop the blot with an ECL substrate and image using a chemiluminescence imager. Following imaging, stain the membrane with methylene blue, wash with ddH_2_O to clean the background, and take final images with a digital camera for documentation.

### 2.13 MeRIP-qPCR

To conduct a meRIP-qPCR (m^6^A RNA Immunoprecipitation followed by quantitative Polymerase Chain Reaction) assay, start by extracting RNA from cells that have been treated under different conditions. Utilize the EpiQuik CUT&RUN m6A RNA Enrichment (MeRIP) Kit from EpigenTek to selectively capture m^6^A-modified RNA fragments. The kit works by cleaving or removing RNA sequences at the ends of target regions that contain m^6^A, while the m^6^A-containing target fragments are pulled down by beads that are coated with m^6^A capture antibodies. After the pull-down, the m^6^A-enriched RNA is released, purified, and eluted. Based on the m^6^A modification enrichment regions identified from MeRIP-seq data, design qPCR primers to specifically target these regions. Finally, perform qPCR using these primers to quantitatively analyze the enrichment of m^6^A at the targeted genomic loci, providing insights into the distribution and abundance of m^6^A modifications within the RNA samples. The MeRIP-qPCR primers used are listed in Table S2.

### 2.14 NR2 synthesis

Starting from 2-chlorostyrene (1) and 2-fluorophenylhydrazine (2), a disubstituted pyrazole compound (3) was constructed by condensation with polyformaldehyde under mixed acid conditions in one step. The compound was then condensed with l-alanine protected by Boc to obtain a stereo mixture 4. The R-shaped compound 5 was successfully obtained through column chromatography, and then the Boc protecting group was removed in an acidic solution to obtain compound 6. Finally, the target product 7 (NR2) was obtained by condensation with adamantane isocyanate, and the molecular crystal of the target product was purified by post-treatment through recrystallization to obtain the molecular crystal of the target product(CCDC Deposition Number:2431247).

### 2.15 Animal studies

6-8-week-old mice male and female mice were purchased from GemPharmatech (Jiangsu, China), including C57BL/6J and nude mice. Nude mice were inoculated with A549-shNLN cells, with 2 × 10^6^ cells per mouse. The tumor cells used were luciferase-stably expressing A549-luci, constructed via pTomo-CMV-luci-T2A-puro lentivirus, selected with 2 μg/mL puromycin for 2 weeks, and verified by dual-luciferase assay. The model was established by subcutaneous injection of 2 × 10^6^ A549-luci cells (150 μL/mouse) into the forelimb axilla. Twelve mice were randomly divided into 2 groups (6 mice/group). The treatment group received intraperitoneal NR2 (25 mg/kg, solvent containing 10 % DMSO, 10 % Solutol HS-15 and saline) from day 6.

Monitoring included mouse status, subcutaneous tumor volume, body weight, and in vivo imaging (150 mg/kg D-luciferin potassium salt i.p., detected 10–15 min later, analyzed by Living Image software). All animals were housed in a specific pathogen free (SPF) facility with 24-hr access to food and water, and with consistent room temperatures (20°C-26 °C) and humidity (40 %–70 %). Animal experiments in this study were approved by Research Ethics Committee of West China Hospital Sichuan University. Mice were euthanized by cervical dislocation under anesthesia. Bioluminescent imaging was acquired with IVIS Spectrum (PerkinElmer).

In the construction of a *KRAS*^G12D^/*P53*^−/−^ primary lung cancer mouse model, eight-week-old male C57 mice were used, with *P53* silenced and *KRAS*^G12D^ overexpressed under the control of the carbonic anhydrase II promoter. The mice were infected in the lungs with a virus solution through tracheal intubation, and then randomly divided into two groups of four for treatment with NR2, which was administered via tail vein injections starting on the 6th day, twice every two days, at a dose of 25 mg/kg per mouse. Due to the invisibility of tumors and the challenges of tail vein injections, the treatment was stopped after seven administrations, and micro CT scans were used to monitor lung tumor progression before endpoint sampling.

### 2.16 Organoid culture and staining

Organoid Culture: *KRAS*^G12D^/*P53*^−/−^ primary mouse lung cancer cells and primary human lung cancer specimens were processed for organoid culture. Isolated cells were resuspended in Matrigel at a density of 5 × 10^4^ cells/100 μL and plated as 50 μL droplets in 24-well plates, which were allowed to solidify at 37 °C for 15 min. Organoids were cultured in Advanced DMEM/F12 supplemented with N2, B27, HEPES, N-acetylcysteine, EGF, FGF2, R-spondin 1, Noggin, and Y-27632, with medium refreshed every 2–3 days. Cultures were passaged every 7–10 days via mechanical dissociation and re-embedding in Matrigel, and maintained at 37 °C in a 5 % CO_2_ incubator.

BODIPY C11 Staining for Lipid Peroxidation: Organoids were treated with vehicle (DMSO, 72 h), 25 μM NR2 (72 h), or 10 μM Ferrostatin-1 (Fer-1, 2 h pretreatment) followed by co-incubation with 25 μM NR2 (72 h). After treatment, organoids were stained with 5 μM BODIPY 581/591C11 at 37 °C for 30 min, washed with PBS, and imaged using the Operetta High-Content Screening System (PerkinElmer), a confocal microscope platform. Oxidized lipids (green, ex/em: 488/510 nm) and non-oxidized lipids (red, ex/em: 581/591 nm) were detected.

Calcein-AM/Hoechst/PI Staining for Cell Viability: Following treatment, organoids were stained with 2 μM Calcein-AM, 10 μg/mL Hoechst 33342, and 4 μM propidium iodide (PI) at 37 °C for 30 min, washed with PBS, and imaged using the Operetta High-Content Screening System (PerkinElmer). Live cells (green, ex/em: 490/515 nm; Calcein-AM^+^), dead cells (red, ex/em: 535/617 nm; PI^+^), and all nuclei (blue, ex/em: 350/461 nm; Hoechst^+^) were identified.

### 2.17 Statistical analysis

The two tailed unpaired Student's test was used to compare mean values between two groups. ANOVA was used to analyze potential differences for multi-group comparisons with continuous variables. Kaplan–Meier survival curves were compared using the log-rank test to assess survival differences between groups. Statistical analysis was conducted using GraphPad Prism version 8.3 software (GraphPad; La Jolla, CA,USA). All the experiments were repeated at least three times with triplicates unless stated otherwise. All tests were two sided, and *P* values < 0.05 were considered to be statistically significant.

## 3 Results

### 3.1 Proteomic analysis reveals elevated NLN in non-small cell lung cancer tissues and associated with poor prognosis

**Fig. 1:**
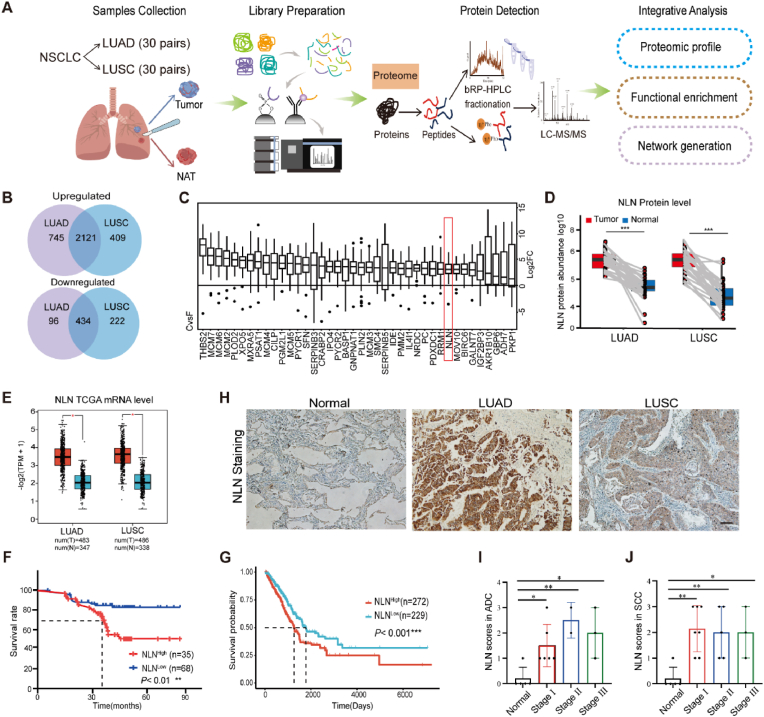
NLN is highly expressed in non-small cell lung cancer. (A) Schematic representation of the label-free quantitative proteomics analysis conducted on 30 cases of lung adenocarcinoma (LUAD) and 30 cases of lung squamous cell carcinoma (LUSC), along with their corresponding adjacent non-cancerous tissues (NAT). (B) Venn diagram displaying the integration of proteomic alterations observed in LUAD and LUSC, identifying upregulated and downregulated proteins. (C) Top 40 significantly different proteins. (D) Absolute quantification analysis of NLN in lung adenocarcinoma and lung squamous cell carcinoma proteomes. (E) Analysis of NLN mRNA level changes in lung cancer using TCGA data. (F) Kaplan-Meier survival curves for lung cancer patients stratified by NLN protein expression levels. (G) Kaplan-Meier survival curves for lung cancer patients stratified by NLN mRNA expression levels. (H) Immunohistochemical analysis of NLN expression in tumor tissues and distal control tissues from patients with lung adenocarcinoma and squamous cell carcinoma. Scale bar: 50 μm. (I, J) Staining intensity scores indicate significantly elevated NLN expression in both types of lung cancer tissues compared to normal lung tissues. Scale bar: 100 μm. Error bars represent standard error of the mean (SEM). ∗*P* < 0.05, ∗∗*P* < 0.01, ∗∗∗*P* < 0.001.

To identify and screen potential new targets for lung cancer treatment, we performed a label-free quantitative proteomics analysis on 30 cases of lung adenocarcinoma and 30 cases of lung squamous cell carcinoma, along with their corresponding adjacent tissues (Fig. 1A). Following a differential protein analysis (Fig. S1A and B), we integrated the proteomic alterations observed in lung adenocarcinoma and squamous cell carcinoma, identifying 2121 upregulated and 434 downregulated proteins (Fig. 1B). By ranking these proteins according to the magnitude of their significant differential expression between lung cancer and adjacent tissues, we identified the top 40 protein factors, which predominantly included enzymes and transcription factors (Fig. 1C). Through an analysis and literature review of these differentially expressed proteins, Neurolysin (NLN) emerged as a particularly noteworthy novel target. While NLN is recognized for its oncogenic role in acute myeloid leukemia (AML), its involvement in solid tumors, including lung cancer, has not been extensively studied [10]. Further comparative analysis of paired samples revealed a significant upregulation of NLN protein expression in both lung adenocarcinoma and squamous cell carcinoma (Fig. 1D). Additionally, utilizing data from The Cancer Genome Atlas (TCGA), we observed that NLN is also highly expressed at the mRNA level in lung adenocarcinoma and squamous cell carcinoma (Fig. 1E). Furthermore, an integrative analysis of proteomic and TCGA transcriptomic data revealed that NLN expression is significantly elevated in tumor tissues of patients with lung adenocarcinoma and squamous cell carcinoma when compared to normal tissues, taking into account both transcript and protein expression levels (Fig. S1C and D). Expression analysis across different stages of lung cancer progression using TCGA data indicated that NLN is significantly upregulated in stage I of both lung adenocarcinoma and squamous cell carcinoma, with a trend of increasing expression as the stage advances in lung adenocarcinoma (Fig. S1E and F).

In a research paper published in 2020, He. et al. conducted the first large-scale, high-throughput, systematic proteomic study of lung adenocarcinoma, identifying several molecular features associated with prognosis [12]. By leveraging the prognostic information from this cohort alongside data from The Cancer Genome Atlas (TCGA), Kaplan-Meier survival analysis indicated that high expression levels of NLN are significantly correlated with poor prognosis in lung cancer (*P* < 0.001) (Fig. 1F and G). To validate the expression of NLN in lung cancer prior to examining its role in the development of human lung cancer, immunohistochemistry was conducted on tumor tissues and distal control tissues from 11 patients with lung adenocarcinoma and 15 patients with lung squamous cell carcinoma. The staining intensity was scored, revealing that NLN expression was significantly elevated in both lung adenocarcinoma and lung squamous cell carcinoma tissues compared to normal lung tissues (Fig. 1H). Further statistical analysis revealed that NLN levels were significantly elevated in early-stage (stage I) lung cancer, aligning with the results obtained from TCGA data analysis (Fig. 1I and J). Additionally, NLN staining was performed on tumor tissues from a *KRAS*^G12D^/*P53*^−/−^ primary mouse lung cancer model, as well as control tissues, demonstrating high NLN expression in the lung cancer lesions of the mouse model (Fig. S1G). These findings suggest that NLN is strongly correlated with the development of lung cancer and may serve as a potential therapeutic target.

### 3.2 NLN facilitates tumorigenicity of lung cancer in vitro and in vivo

**Fig. 2:**
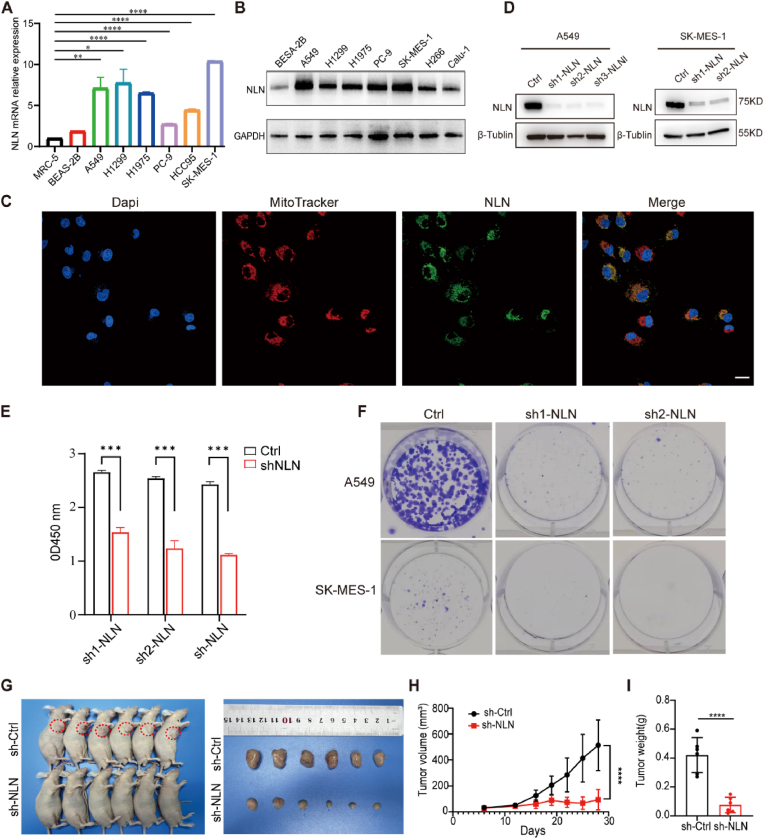
Functional changes in lung cancer cells following NLN knockdown. (A) qRT-PCR analysis of *NLN* mRNA expression levels in multiple cell lines. MRC-5 (human embryonic lung cells), BESA-2B cells (human bronchial epithelial cells), A549 cells (human lung alveolar basal epithelial cells), H1299 cells (human lung cancer cells), H1975 cells (human lung adenocarcinoma cells), PC-9 cells (human lung cancer cells), HCC95 (human lung squamous cell carcinoma), SK-MES-1 cells (lung squamous cell carcinoma). (B) Western blot analysis of NLN protein expression levels in various cell lines. H266 cells, Calu-1 cells (lung adenocarcinoma cells). (C) A549-NLN-mEGFP cell MitoTracker staining. Merge, the three colors channels merge. Scale bar: 100 μm. (D) Western blot analysis of the knockdown efficiency in stable A549 and SK-MES-1 cell lines constructed with three or two sh-NLN sequences. (E) CCK8 assay showing the proliferation ability of A549-shNLN cell lines. (F) Colony formation assay after stable knockdown of NLN in A549 and SK-MES-1 cells. (G) Photographs of nude mouse xenograft tumors. Left panel shows the tumors before removal; right panel shows the tumors after removal. (H) Growth curve of xenograft tumor volumes. (I) Statistical analysis of xenograft tumor weights. Error bars represent standard error of the mean (SEM). ∗*P* < 0.05, ∗∗*P* < 0.01, ∗∗∗*P* < 0.001, ∗∗∗∗*P* < 0.0001.

We next evaluated the expression levels of *NLN* in various lung cancer cell lines through quantitative reverse transcription polymerase chain reaction (qRT-PCR) and Western blot analysis. In comparison to the normal lung cell lines MRC-5 and BEAS-2B, NLN exhibited significantly higher expression across multiple lung cancer cell lines, with particularly elevated levels observed in the adenocarcinoma cell line A549 and the squamous cell carcinoma cell line SK-MES-1 (Fig. 2A and B). To comprehensively assess the expression landscape of *NLN* in NSCLC, we extended our analysis to a large panel of ∼142 NSCLC cell lines from the DepMap database (Public 25Q3). This unbiased analysis revealed substantial heterogeneity in *NLN* mRNA expression, with levels varying widely from low to high across the cell line panel (Fig. S2A). In summary, our experimental findings corroborate the proteomic results, demonstrating that NLN is highly expressed in both lung cancer patient tissues and a subset of lung cancer cell lines. This observation suggests a potential role for NLN in promoting lung carcinogenesis within this distinct subgroup.

Following gene translation into proteins, the subcellular localization of these proteins can provide valuable insights into their functional roles. Previous studies reported that NLN is located in mitochondria and is also secreted into the circulation [13]. To confirm whether NLN localizes to the mitochondria, we selected the A549 cell line, known for its high expression levels of NLN, to conduct immunofluorescence assays to examine NLN's localization. Our findings revealed that NLN is primarily localized in the cytoplasm, displaying a slender, filamentous distribution (Fig. S2B). To more effectively visualize the subcellular distribution of NLN under physiological conditions, we fused the C-terminus of NLN with green fluorescent protein (GFP) and successfully established A549-NLN-mEGFP cells. We then stained these cells with Mito-Tracker, enabling us to observe mitochondrial localization concurrently. The results demonstrated a significant degree of colocalization between the fluorescence signals of NLN and mitochondria, thereby confirming that NLN is predominantly localized to the mitochondria within the cell (Fig. 2C). These findings also suggest that targeting NLN may cause mitochondrial damage.

To explore the oncogenic role of NLN in cell growth and migration, we evaluated the effects of NLN on cell phenotypes. Stable knockdown was achieved by infecting cells with lentiviral constructs expressing different shRNAs. Knockdown with each shRNA reduced NLN protein and mRNA levels by approximately 90 % (Fig. 2D; Fig. S2C and D). In the CCK-8 assays, likewise, cell growth was significantly inhibited in sh-NLN compared with Ctrl cells (Fig. 2E). NLN knockdown also inhibited colony formation of A549 and SK-MES-1 cells (Fig. 2F). We also investigated the biological consequences of NLN knockdown through the use of small interfering RNAs (siRNAs). Each of the three siRNAs demonstrated high efficiency, resulting in a marked reduction in NLN expression (Fig. S2E and F). Through Transwell assays, we observed that the suppression of NLN expression significantly attenuated the metastatic potential of lung cancer cells (Fig. S2G).

To further investigate the tumorigenic function of NLN in vivo, we established a doxycycline (DOX)-inducible conditional NLN knockdown cell model, A549-Tet-shNLN, and conducted subcutaneous xenograft tumor experiments in nude mice. Consistent with the in vitro results, a reduction in tumor burden was observed following the knockdown of NLN (Fig. 2G). Both the tumor volume and weight showed remarkable reduction in the shNLN group compared with the shCtrl group, indicating that stable depletion of NLN effectively inhibited tumor growth in vivo (Fig. 2H and I). Immunohistochemical staining was conducted on subcutaneous tumor tissues from both the control group (sh-Ctrl) and the NLN knockdown group (sh-NLN). The results demonstrated a significant reduction in NLN expression levels within the tumor tissues of the sh-NLN group (Fig. S3A and B), indicating that the observed attenuation in tumor growth is likely a result of NLN suppression. Additionally, Ki67 staining was utilized to assess the proliferative status at the molecular pathological level, revealing a significant decrease in Ki67 expression following NLN knockdown (Fig. S3C). Taken together, our results demonstrate that NLN facilitates lung cancer tumorigenesis both in vitro and in vivo.

### 3.3 NLN inhibits ferroptosis in lung cancer cells

**Fig. 3:**
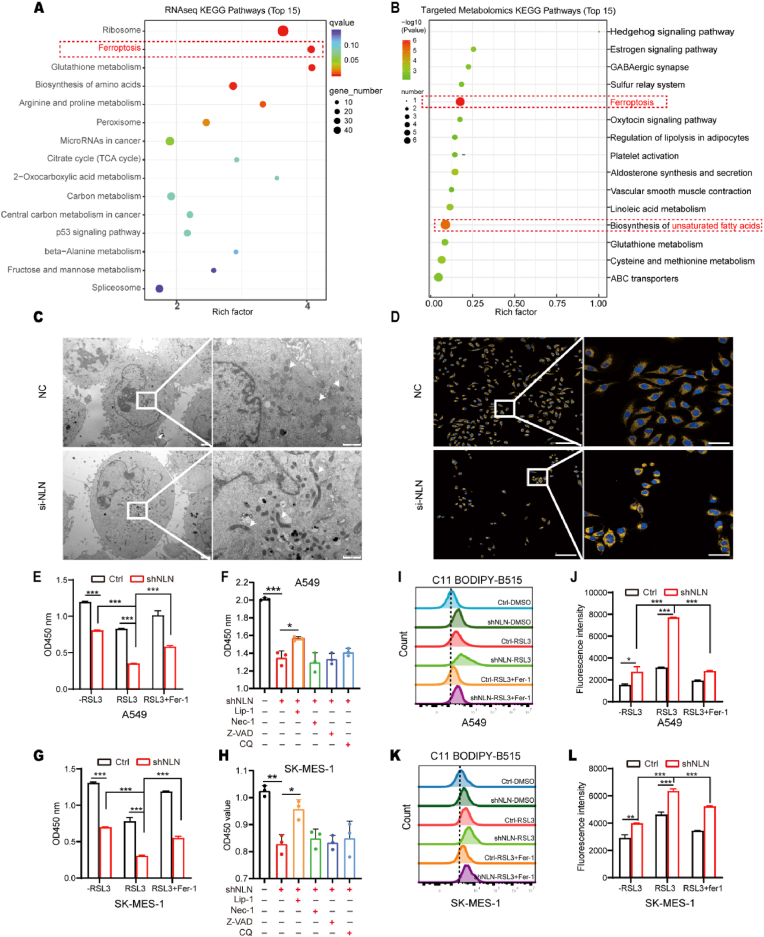
NLN inhibition induces ferroptosis in lung cancer cells. (A) Scatter plot of KEGG pathway enrichment based on differentially expressed transcripts from third-generation sequencing following stable knockdown of NLN. Larger enrichment factors indicate a more significant enrichment of differentially expressed transcripts in that pathway. (B) Bubble plot of KEGG pathway enrichment. Top 15 significant pathways selected based on p-values. (C) Transmission electron microscopy images of A549 cells with NLN knockdown showing mitochondrial morphology (white arrows). Scale bars: 1 μm (left panel), 2 μm (right panel). (D) Mitochondrial staining with Mito Tracker in A549 cells post-NLN knockdown. Blue (DAPI): nucleus; yellow: mitochondria. Scale bars: 200 μm (left panel) and 50 μm (right panel). (E) Cell viability assay (CCK8) of control A549 (ctrl) and NLN knockdown A549 cells (shNLN). Cells were pretreated with or without Ferrostatin-1 (Fer-1, 2 μM) for 2 h, followed by co-treatment with or without RSL3 (1 μM) for 72 h. (F) shNLN A549 cells viability were assessed using the CCK8 assay treated with various cell death inhibitors: Liproxstatin-1 (Lip-1), Necrostatin-1 (Nec-1), Z-VAD-FMK (Z-VAD), and Chloroquine (CQ). (G) CCK8 assay analyzed the viability of SK-MES-1 cells (ctrl and shNLN). Cells were pretreated with or without Ferrostatin-1 (Fer-1, 2 μM) for 2 h, followed by co-treatment with or without RSL3 (2 μM) for 72 h. (H) CCK8 assay analyzed the viability of sh-NLN SK-MES-1 cells treated with various cell death inhibitors. (I) Flow cytometric analysis of BODIPY™ 581/591C11 staining in A549 cells. (J) Mean fluorescence intensity plots of BODIPY™ 581/591C11 show the intracellular lipid peroxidation in A549 cells. (K) Flow cytometric analysis of BODIPY™ 581/591C11 staining in SK-MES-1 cells. (L) Mean fluorescence intensity plots of BODIPY™ 581/591C11 staining in SK-MES-1 cells. ∗*P* < 0.05, ∗∗*P* < 0.01, ∗∗∗*P* < 0.001.

We further investigated the mechanism by which NLN exerts its oncogenic function in lung cancer. Initially, we employed a stably knocked-down shNLN cell line for Nanopore sequencing, a third-generation sequencing technology that utilizes single-molecule real-time sequencing through nanopores, facilitating the collection of comprehensive genomic information related to cancer [14,15]. Differential transcript abundance was visualized via a heatmap (Fig. S4A), revealing distinct expression profiles between the control group (shCtrl) and the NLN knockdown group (sh-NLN). Pathway enrichment analysis indicated significant upregulation of ribosome-related pathways, ferroptosis pathways, and glutathione metabolism pathways, alongside a notable downregulation of the spliceosome pathway, with the ferroptosis pathway being particularly enriched (Fig. 3A). This suggests a close relationship between NLN and ferroptosis, a regulated form of cell death dependent on iron, characterized by uncontrolled lipid peroxidation and subsequent rupture of the plasma membrane [5]. Given that ferroptosis can be modulated by various metabolic pathways involving lipids, iron, amino acids, and glutathione metabolism [16], we subsequently performed high-throughput targeted metabolomics analysis using the H650-targeted metabolomics analysis. The results indicated a significant increase in differential metabolites, such as polyunsaturated fatty acids like arachidonic acid, alongside a significant decrease in cysteine following NLN knockdown (Fig. S4B). KEGG pathway enrichment analysis further revealed significant upregulation of pathways associated with ferroptosis and polyunsaturated fatty acid synthesis (Fig. 3B). These metabolomics findings reinforce the notion that the ferroptosis pathway is critical in the regulatory mechanism of NLN.

To investigate the putative role of NLN in the ferroptosis, we examined the characteristic indicators associated with ferroptosis. Morphological characteristics of cells undergoing ferroptosis include shrunken mitochondria with increased membrane density [17,18]. Using transmission electron microscopy, we observed that lung cancer A549 cells with NLN knockdown exhibited both mitochondrial shrinkage and increased membrane density (Fig. 3C). Following staining with the mitochondrial dye Mito Tracker and subsequent imaging, we noted a shift in the mitochondrial staining pattern from an elongated filamentous form to a retracted form, localized near the nucleus after NLN knockdown (Fig. 3D). These findings indicate that the suppression of NLN expression may induce ferroptosis in lung cancer cells. RSL3, a ferroptosis inducer, selectively inhibits glutathione peroxidase 4 (GPX4). By blocking GPX4's function, it disrupts the cell's antioxidant defense, leading to lipid peroxide accumulation and ferroptotic cell death [7]. To confirm that the cell death induced by NLN knockdown is indeed ferroptosis rather than another form of cell death, we assessed the sensitivity of lung cancer cells to RSL3-induced ferroptosis following NLN suppression. Our results demonstrated that the inhibition of NLN significantly enhanced RSL3-induced cell death, and pretreatment with Ferrostatin-1 significantly reversed the enhanced sensitivity of NLN-knockdown cells to RSL3 (Fig. 3E–G). To further validate these findings, we performed detailed dose-response analyses which confirmed a significantly decreased IC_50_ value for RSL3 upon NLN knockdown (Fig. S4C and D), providing quantitative evidence for enhanced ferroptosis sensitivity. The specificity of this effect was confirmed by the observation that this inhibition was reversed by the ferroptosis inhibitor Liproxstatin-1, but not by inhibitors targeting other cell death pathways, such as the necroptosis inhibitor Nec-1, the apoptosis inhibitor Z-VAD, or the autophagy inhibitor CQ (Fig. 3F–H). The lack of rescue by Z-VAD-FMK (apoptosis inhibitor) and Necrostatin-1 (necroptosis inhibitor) under our specific experimental conditions suggests that NLN knockdown does not significantly sensitize cells to these alternative death modalities, though their general competence for these pathways was not formally tested here. Collectively, These data indicate that ferroptosis as the primary mode of cell death enhanced by NLN inhibition under our experimental conditions.

Intracellular lipid peroxidation is a hallmark of ferroptosis and serves as a gold standard for its detection. We used BODIPY™ 581/591C11, a fluorescent probe that shifts from red to green fluorescence upon lipid peroxidation, to visualize intracellular lipid peroxidation in A549 and SK-MES-1 cells with NLN knockdown or control. The results showed a significant increase in lipid peroxidation in NLN-knockdown cells (Fig. 3I–L). To establish a positive control, RSL3 was used to induce lipid peroxidation. Notably, NLN-knockdown cells exhibited significantly higher RSL3-induced lipid peroxidation compared to control cells, and this effect was markedly attenuated by Ferrostatin-1 pretreatment in both groups (Fig. 3I–L). These results confirm that the observed lipid peroxidation is ferroptosis-specific and further indicate that NLN deficiency enhances sensitivity to ferroptosis induction. Additionally, we used the Liperfluo fluorescent probe, which specifically detects lipid peroxides in the plasma membrane of living cells [19,20], to examine membrane lipid peroxidation. Consistently, NLN knockdown significantly increased plasma membrane lipid peroxidation in A549 cells (Fig. S4E and F), further supporting that NLN deficiency promotes ferroptosis-associated lipid peroxidation in both intracellular and membrane compartments. Reactive oxygen species (ROS) are recognized as principal mediators of ferroptosis and serve as hallmark biomarkers. Given that mitochondria are a major source of intracellular ROS [11], we employed the CellROX™ Deep Red reagent to assess general intracellular oxidative stress. Our results indicated a significant increase in ROS levels within the cells following NLN knockdown. It should be noted that the exact nature of the ROS detected remains to be fully determined, as this probe measures overall oxidative stress without distinguishing between specific ROS subtypes. By integrating these findings with the observed rise in lipid peroxidation, we further substantiate the correlation between NLN knockdown and ferroptosis, suggesting that NLN suppression results in elevated levels of both intracellular lipid peroxidation and ROS (Fig. S4G and H). Intracellular accumulation of ferrous iron (Fe^2+^) is a prerequisite for ferroptosis. Fe^2+^ reacts with hydrogen peroxide (H_2_O_2_) through the Fenton reaction, generating highly reactive hydroxyl radicals, which are the most potent among reactive oxygen species (ROS) and can induce peroxidation of both unsaturated and saturated fatty acids in membrane lipids [21]. The xCT-GPX4-GSH axis represents a critical system within the cell that combats oxidative stress and is a key component of ferroptosis resistance [22,23]. Consequently, intracellular levels of GSH and cystine uptake serve as characteristic indicators of ferroptosis. We assessed intracellular ferrous iron levels using the FerroOrange fluorescent probe and measured intracellular GSH levels and cystine uptake through assay kits. The results demonstrated a significant increase in intracellular ferrous iron, a decrease in intracellular glutathione levels, and a reduction in cystine uptake (Fig. S4I–K). Collectively, all of these findings confirm that the knockdown of NLN induces ferroptosis in lung cancer cells.

### 3.4 NLN knockdown induces ferroptosis in lung cancer cells via degradation of GPX4 mRNA

**Fig. 4:**
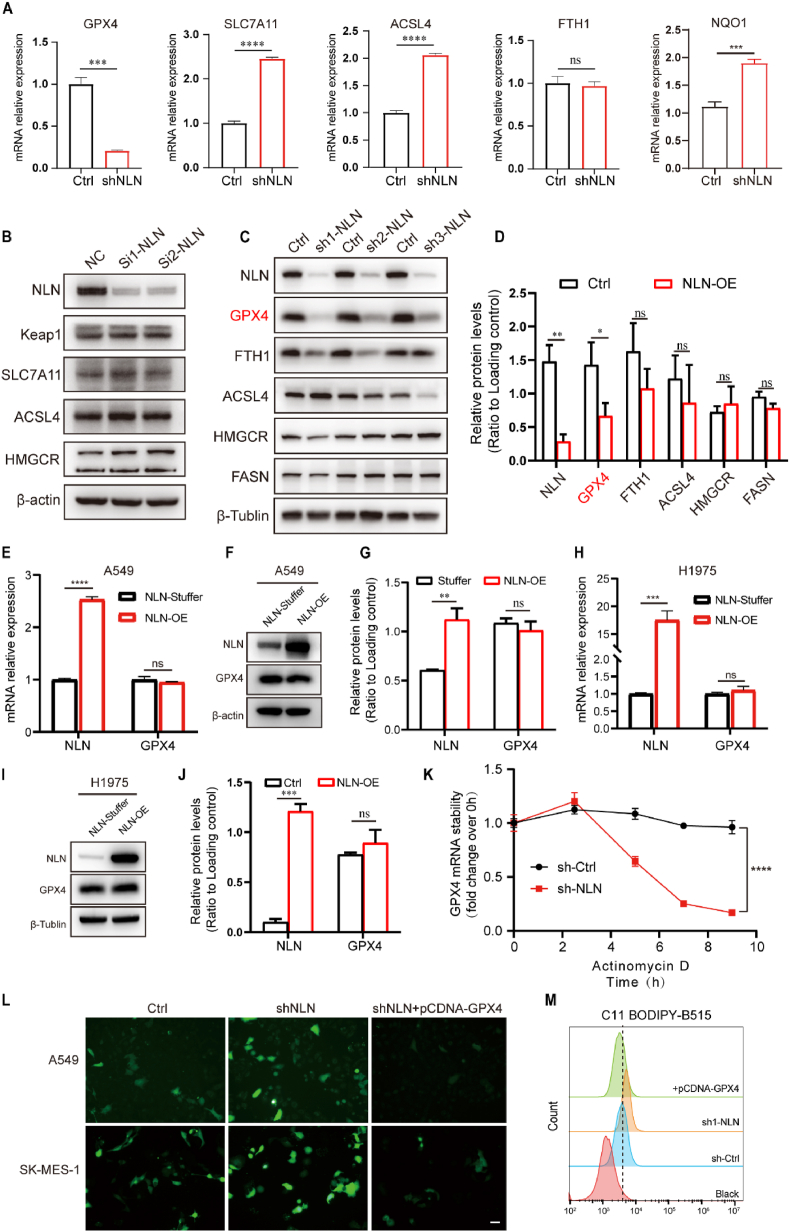
NLN knockdown induces ferroptosis in lung cancer by degrading GPX4 mRNA. (A) qRT-PCR analysis of ferroptosis related factors following stable knockdown of NLN. (B) Western blot analysis of ferroptosis related factors following siRNA-mediated knockdown of NLN. (C) Western blot analysis of ferroptosis related factors following stable knockdown of NLN. GPX4 (glutathione peroxidase 4), SLC7A11 (solute carrier family 7 member 11), ACSL4 (acyl-CoA synthetase long chain family member 4), FTH1 (ferritin heavy chain 1), FTL (ferritin light chain), PTGS2 (prostaglandin-endoperoxide synthase 2), HMGCR (3-hydroxy-3-methylglutaryl-coenzyme A reductase), FASN (fatty acid synthase). (D) Relative quantification of western blots shown in (C). (E) qRT-PCR analysis of GPX4 mRNA levels in A549 cells following stable overexpression of NLN. (F) Western blot analysis of GPX4 protein levels in A549 cells following stable overexpression of NLN. (G) Relative quantification of western blots shown in (F). (H) qRT-PCR analysis of GPX4 mRNA levels in H1975 cells following stable overexpression of NLN. (I) Western blot analysis of GPX4 protein levels in H1975 cells following stable overexpression of NLN. (J) Relative quantification of western blots shown in (I). (K) Actinomycin D treatment to assess mRNA stability (normalized to 0h). (L) BODIPY™ 581/591C11 staining of A549 and SK-MES-1 cells to assess lipid peroxidation under different treatment conditions. Scale bar: 100 μm. (M) Flow cytometry analysis of fluorescence intensity changes in BODIPY™ 581/591C11-stained A549 cells under different treatment conditions. X-axis represents average fluorescence intensity. Error bars represent standard error of the mean (SEM). ∗*P* < 0.05, ∗∗*P* < 0.01, ∗∗∗*P* < 0.001, ∗∗∗∗*P* < 0.0001.

Following the determination that *NLN* knockdown induces ferroptosis in lung cancer cells, we sought to elucidate the potential mechanisms by which NLN regulates this process. Utilizing quantitative reverse transcription polymerase chain reaction (qRT-PCR) and Western blot analysis, we evaluated various factors implicated in ferroptosis. Our results indicated that at the mRNA level, the expression of *GPX4* was significantly reduced. Interestingly, the upregulation of *SLC7A11* and *NQO1* may be attributed to a downstream feedback mechanism that counters the suppression of *GPX4*, while *ACSL4,* which is associated with the synthesis of polyunsaturated fatty acids, showed increased expression. Additionally, no significant variation was observed in *FTH1*, a factor involved in iron metabolism (Fig. 4A). Protein level assessments corroborated these findings, revealing a marked decrease in GPX4, with no substantial changes in the levels of *KEAP1*, *SLC7A11*, *ACSL4*, *HMGCR*, and *FASN* (Fig. 4B–D). By integrating the alterations observed at both the mRNA and protein levels, we propose that following NLN knockdown, GPX4 emerges as the most critical downstream factor in the NLN-mediated induction of ferroptosis in lung cancer cells, which is also one of the key components within the ferroptosis pathway. However, in an intriguing finding, the stable overexpression of NLN in A549 and H1975 cells did not lead to a significant increase in GPX4 expression, neither at the mRNA nor at the protein level (Fig. 4E–J). This observation implies that NLN may not regulate GPX4 expression at the transcriptional level. Consequently, we hypothesized that NLN could exert its regulatory effects on GPX4 at the post-transcriptional level. To investigate this hypothesis, we conducted an mRNA stability assay using Actinomycin D, which evaluates the rate of mRNA degradation and stability. Our results indicated that in the NLN knockdown group, GPX4 mRNA underwent rapid degradation after 2 h, showing a significant divergence from the control group, and was nearly completely degraded by 9 h. This suggests that NLN regulates GPX4 expression by influencing the stability of its mRNA (Fig. 4K). Subsequently, we rescued the loss of GPX4 caused by NLN, through overexpression of GPX4, alongside BODIPY™ 581/591C11 staining to assess lipid peroxidation. The results indicated that the downregulation of NLN led to increased levels of intracellular lipid peroxidation. Importantly, the overexpression of GPX4 in the context of NLN knockdown significantly reduced intracellular lipid peroxidation, thereby providing protection against ferroptosis (Fig. 4L and M). This finding supports the conclusion that NLN modulates the ferroptosis process through the regulation of GPX4 levels.

### 3.5 NLN knockdown decreases GPX4 mRNA stability in an m^6^A-dependent manner

**Fig. 5:**
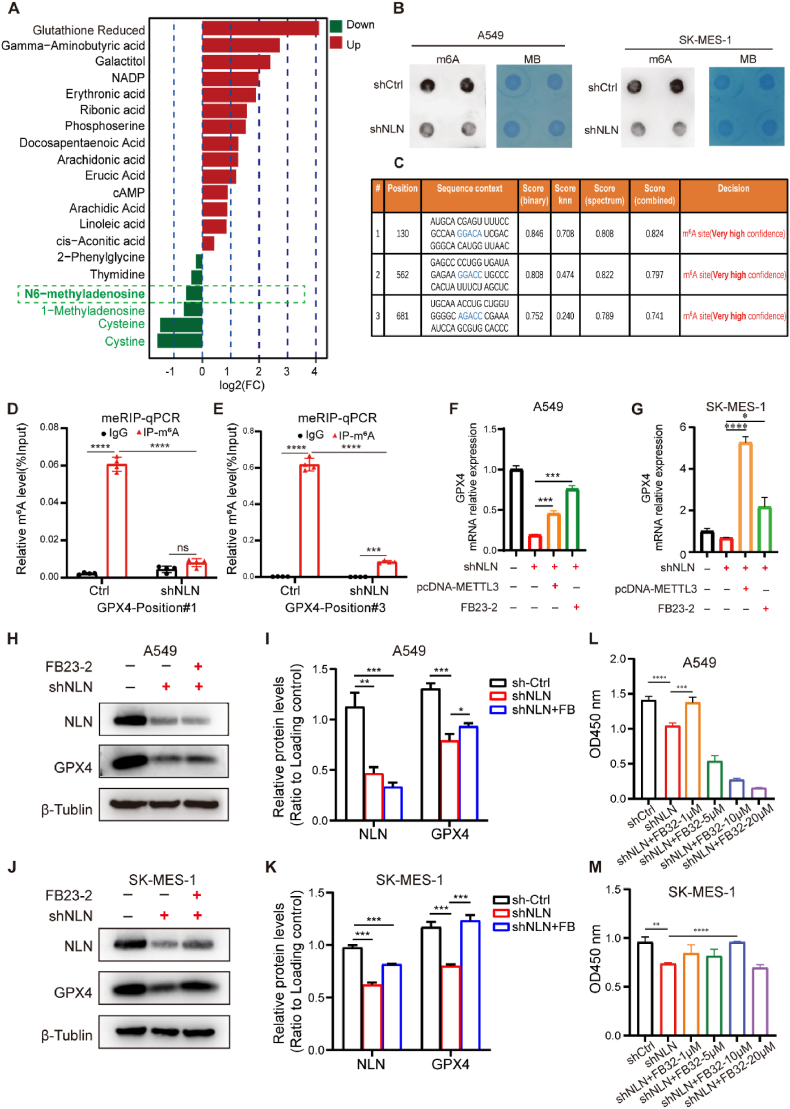
Knockdown of NLN inhibits m^6^A modification of GPX4. (B) Fold change analysis of significantly different metabolites. (B) RNA dot blot analysis of m^6^A modification levels in A549 and SK-MES-1 cells following NLN knockdown. (C) Predicted m^6^A modification sites on *GPX4* mRNA. (D, E) MeRIP-qPCR analysis demonstrating significant reduction in m^6^A modification at positions #1 and #3 of *GPX4* mRNA after NLN knockdown. (F) qRT-PCR analysis of *GPX4* mRNA levels in A549 cells overexpressing *METTL3* and inhibiting FTO activity. (G) qRT-PCR analysis of GPX4 mRNA levels in SK-MES-1 cells overexpressing *METTL3* and inhibiting FTO activity. (H) Western blot analysis of GPX4 protein levels in A549 cells following FTO activity inhibition. (I) Relative quantification of western blots shown in (H). (J) Western blot analysis of GPX4 protein levels in SK-MES-1 cells following FTO activity inhibition. (K) Relative quantification of western blots shown in (J). (L, M) The cell viability of A549 and SK-MES-1 cells following treatment with varying concentrations of FB23-2 indicated by CCK8 assay results. Error bars represent standard error of the mean (SEM). ∗*P* < 0.05, ∗∗*P* < 0.01, ∗∗∗*P* < 0.001, ∗∗∗∗*P* < 0.0001.

Following the discovery that NLN modulates the post-transcriptional mRNA stability of *GPX4* to influence its expression, we further investigated the mechanisms by which NLN regulates *GPX4* mRNA stability. Metabolomic profiling revealed that NLN depletion resulted in decreased cellular levels of N1-methyladenosine (m^1^A) and N6-methyladenosine (m^6^A) (Fig. 5A). While m1A modification is associated with protein translation, m^6^A modification is closely linked to mRNA stability [24,25]. Therefore, we propose that reduced NLN levels lead to diminished m^6^A modification of *GPX4*, resulting in *GPX4* mRNA instability and subsequently triggering ferroptosis in lung cancer cells. To explore this hypothesis, we utilized qPCR to examine changes in genes related to m^6^A modification and found a significant downregulation of *METTL3* and *IGF2BP2* following NLN knockdown (Fig. S5A), suggesting a potential correlation between NLN and intracellular m^6^A levels. Utilizing the RNA dot blot technique, we assessed the global levels of m^6^A modification on cellular RNA. Our findings revealed that the knockdown of NLN in both A549 and SK-MES-1 cells led to a decrease in m6A modification levels (Fig. 5B). Furthermore, using an m^6^A RNA Methylation Quantification Kit for detection, the results confirmed a significant reduction in intracellular m^6^A levels in A549 cells following the knockdown of NLN (Fig. S5B).

To investigate whether NLN directly modulates the mRNA modification levels of *GPX4*, we predicted the sites on *GPX4* mRNA most susceptible to m^6^A modification using the SRAMP website, as illustrated in Fig. 5C. Positions #1 to #3 were identified as the most likely sites for m^6^A modification on *GPX4* mRNA. Subsequently, we conducted meRIP-qPCR which can verify the modification level of specific modification sites (in the form of a small region) on a target gene to assess changes in m^6^A modification levels at these sites following the knockdown of NLN. The findings indicated that the m^6^A modification levels at positions #1 and #3 of *GPX4* mRNA were significantly reduced after NLN knockdown (Fig. 5D and E). Position #2 could not be evaluated due to undetectable levels of m^6^A modification, thereby confirming that the knockdown of NLN directly downregulates the m^6^A modification levels of *GPX4* mRNA. After demonstrating that the knockdown of NLN can reduce the m^6^A modification level of *GPX4* mRNA, we proceeded to investigate the hypothesis that m^6^A modification influences the expression of GPX4. Following the NLN knockdown, we overexpressed the methyltransferase *METTL3* and applied the FTO demethylase inhibitor FB23-2. The results indicated that treatment with FB23-2 for 48 h in A549 cells led to a significant increase in *GPX4* mRNA levels and a significant but small increase in GPX4 protein levels. Consistently, *METTL3* overexpression significantly enhanced *GPX4* levels in A549 cells (Fig. 5F–H, I; Fig. S5C). Parallel experiments in SK-MES-1 cells demonstrated that FB23-2 treatment significantly restored both the mRNA and protein levels of GPX4. Due to the high transfection efficiency in SK-MES-1 cells, the overexpression of *METTL3* resulted in a restoration of GPX4 that even exceeded the control levels (Fig. 5G–J, K; Fig. S5D). These findings highlight that NLN regulates the expression of GPX4 through m^6^A modification.

Knockdown of NLN in A549 and SK-MES-1 cells significantly induced lipid peroxidation, an effect effectively reversed by treatment with FB23-2, an FTO inhibitor, suggesting restoration of m^6^A modification to suppress ferroptosis in NLN-deficient cells (Fig. S5E–H). Control experiments confirm FB23-2 lacks intrinsic anti-ferroptotic activity: it failed to rescue RSL3-induced ferroptosis, whereas the iron chelator DFO successfully mitigated RSL3 effects (Fig. S5E and F). These data collectively demonstrate that NLN regulates ferroptosis through m^6^A modification, as FB23-2 specifically counteracts NLN-mediated ferroptosis by restoring m^6^A-dependent GPX4 regulation independent of direct iron chelation or anti-ferroptotic properties. To further investigate the implications of this regulation on cellular function, treatment of NLN knockdown A549 and SK-MES-1 cells with varying concentrations of FB23-2 demonstrated that a concentration of 1 μM fully restored the viability of A549 cells, while a concentration of 10 μM fully restored the viability of SK-MES-1 cells (Fig. 5L and M). These results reinforce the conclusion that the downregulation of m^6^A modification due to NLN knockdown contributes to the degradation of *GPX4* mRNA, ultimately leading to ferroptosis in these cells.

### 3.6 NR2 as a selective and potent NLN inhibitor exhibits promising anti-lung cancer efficacy in vitro

**Fig. 6:**
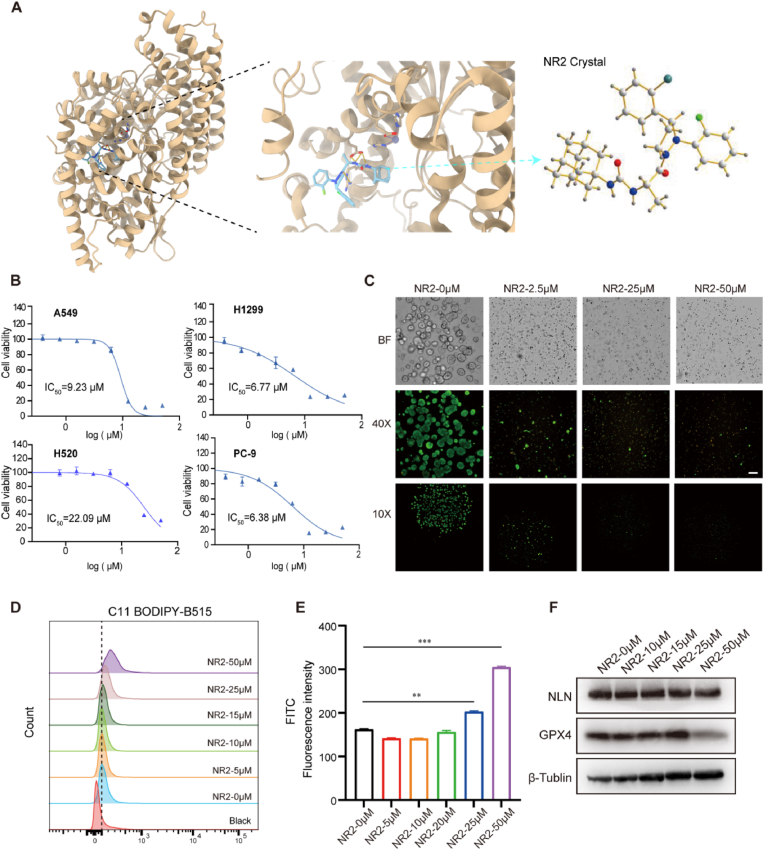
NR2 induces ferroptosis in lung cancer cells in vitro and inhibits malignant functions. (A) Molecular docking results showing the binding pose of NR2 (green) within the active site of NLN (PDB: 8VJU). The binding affinity is indicated as −10.5 kcal/mol (B)In vitro cytotoxicity assays revealing IC_50_ values for NR2 in various lung cancer cell lines. A549 cells (human alveolar basal epithelial cells of lung cancer), H1299 cells (human lung cancer cells), H520 cells (human lung squamous carcinoma cells), and PC-9 cells (human lung adenocarcinoma cells). (C) High-content imaging of lung squamous cell carcinoma organoids treated with different concentrations of NR2. Green fluorescence: live cell staining, yellow fluorescence: dead cell staining. (D) Concentration-dependent increase in intracellular lipid peroxidation in A549 cells treated with NR2, as indicated by BODIPY™ 581/591C11 staining. The x-axis represents mean fluorescence intensity. (E) Statistical graph of mean fluorescence intensity from flow cytometry of A549 cells treated with different concentrations of NR2 after C11 staining. (F) Western blot analysis of GPX4 protein levels in A549 cells treated with different concentrations of NR2. Error bars represent standard error of the mean (SEM). ∗∗*P* < 0.01, ∗∗∗*P* < 0.001.

To further substantiate the clinical significance of NLN as a therapeutic target in NSCLC, we aimed to identify a specific small molecule inhibitor of NLN. The compound 3-[(2S)-1-[(3R)-3-(2-Chlorophenyl)-2-(2-Fluorophenyl)pyrazolidin-1-yl]-1 -oxopropan-2-yl]-1-(adamantan-2-yl) urea, referred to as Neurolysin-R2 (NR2), is an allosteric inhibitor of NLN that has been shown to inhibit the proliferation of acute myeloid leukemia (AML) cells in vitro [10,26]. However, since this small molecule is not commercially available, we synthesized NR2 and confirmed the absolute configuration of its chiral centers through crystal structure analysis (Fig. S6A), with the relevant crystal parameters illustrated in Fig. 6SB. Following crystallization, we performed molecular docking using the recently reported crystal structure of NLN (PDB: 8VJU) [27]. The results indicated that NR2 exhibits a strong binding affinity for NLN, effectively binding near the active center with an affinity of −10.5 kcal/mol (Fig. 6A). Subsequently, we conducted in vitro cytotoxicity assays and determined the IC_50_ values across a panel of lung cancer cell lines. The results demonstrated that the inhibitor exhibits cytotoxic effects on various lung cancer cell lines, with IC_50_ values of 9.23 μM for A549 cells, 6.77 μM for H1299 cells, 22.09 μM for H520 cells, and 6.38 μM for PC-9 cells (Fig. 6B). To further investigate the antitumor effects of NR2 in vitro, we established organoids derived from lung adenocarcinoma and squamous cell carcinoma. After 48-h treatment with varying concentrations of NR2, lung adenocarcinoma and squamous cell carcinoma organoids were dual-stained with Calcein (to identify viable cells) and propidium iodide (PI, to mark dead cells). These analyses revealed that both organoid types exhibited high sensitivity to NR2, with a notable inhibitory effect observed at a concentration of 2.5 μM (Fig. 6C–S6C). These results indicate that NR2 can effectively induce tumor cell death in vitro and demonstrates significant antitumor activity.

We further investigated the ability of NR2 to induce ferroptosis in lung cancer cells. Following treatment with NR2, A549 cells demonstrated a concentration-dependent increase in intracellular lipid peroxidation, with particularly significant effects observed at concentrations of 25 μM and 50 μM (Fig. 6D and E). Microscopic examination of cellular morphology revealed clear signs of cell death at these concentrations, which corresponded with a marked increase in intracellular lipid peroxidation levels, as indicated by green fluorescence (Fig. S6D). Pretreatment with Fer-1 significantly reversed both NR2-induced cell growth inhibition (Fig. S6E) and lipid peroxidation (Fig. S6F and G). These findings suggest that NR2 effectively triggers ferroptosis in lung cancer cells. Additionally, Western Blot analysis of GPX4 protein levels in A549 cells treated with varying concentrations of NR2 confirmed that at a concentration of 50 μM, GPX4 levels were significantly reduced (Fig. 6F). This result indicates that NR2 induces ferroptosis in lung cancer cells via the inhibition of GPX4.

### 3.7 NLN as a promising therapeutic target in lung cancer

**Fig. 7:**
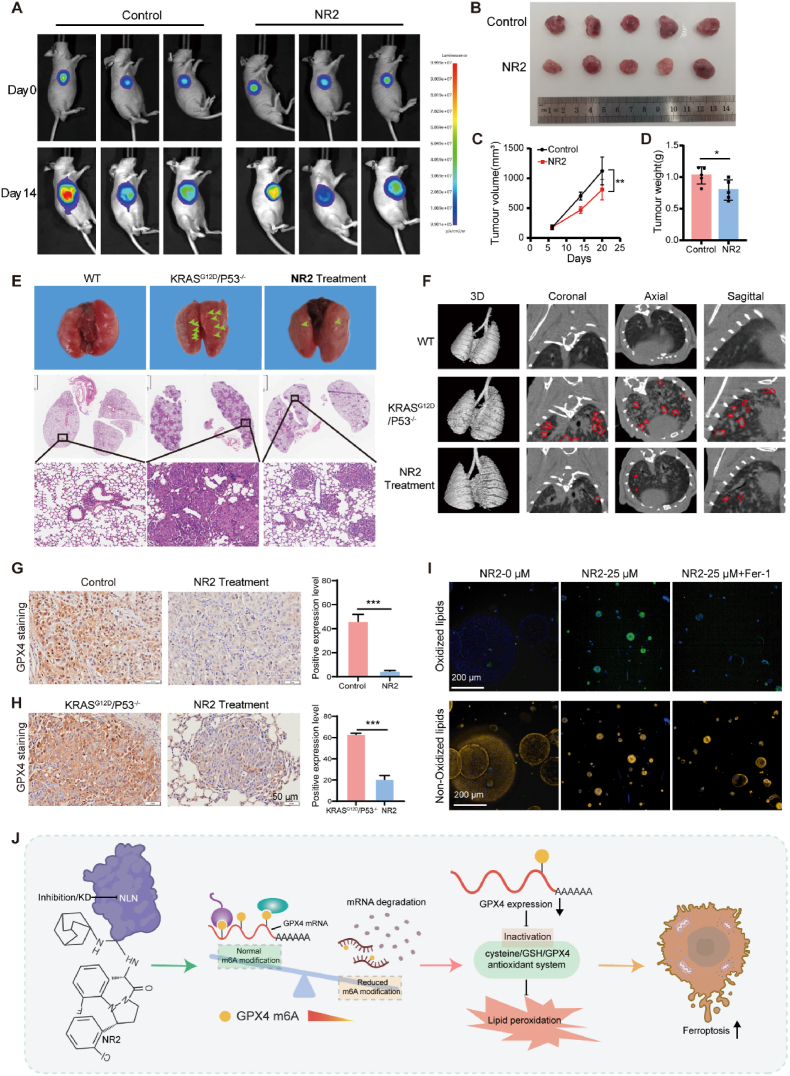
Therapeutic potential of NLN inhibition with NR2 in lung cancer. (A) Live animal imaging monitoring of tumor burden at specific time points in the Control and NR2 treatment groups. (B) Tumor tissue was removed from Control group (Vehicle) and NR2 treatment group mice at the endpoint time and photographed for observation. (C) Volume growth graphs for solvent control (Vehicle) and NR2 dosing groups. (D) Tumor weight statistics of Control group (Vehicle) and NR2 treatment group mice. (E) Surface observation of lung tissues from WT C57BL/6 mice, *KRAS*^G12D^ vehicle group, and *KRAS*^G12D^ NR2 treatment group mice. Observation of lung tissue structure counts using HE staining in different groups of mice. scale bars: 2 mm(upper) and 50 μm (lower). (F) Lung Micro-CT imaging performed after NR2 treatment. WT, wild-type C57 adult mouse; *KRAS*^G12D^/*P53*^−/−^, primary lung cancer model mouse; NR2-Treatment, primary lung cancer model mouse subjected to NR2 treatment; 3D, three-dimensional lung reconstruction; Coronal, coronal plane; Axial, cross-section; Sagittal, sagittal plane. (G). Quantification of immunohistochemical staining intensity showing reduced GPX4 protein levels in subcutaneous tumors after NR2 administration. Scale bars: 50 μm. (H). Quantification of immunohistochemical staining intensity demonstrating decreased GPX4 levels in *KRAS*^G12D^/*P53*^−/−^-driven primary lung cancer mouse models following NR2 treatment. Scale bars: 50 μm. (I). Lipid peroxidation in *KRAS*^G12D^/*P53*^−/−^ mouse lung cancer-derived organoids treated with NR2 and Fer-1. Green fluorescence indicates oxidized lipids, red fluorescence indicates non-peroxidized lipids, and blue fluorescence (Hoechst staining) marks cell nuclei. Scale bars: 200 μm. (J) The schematic of how NLN regulates GPX4 mRNA degradation and ferroptosis in lung cancer. Error bars represent standard error of the mean (SEM). ∗*P* < 0.05, ∗∗*P* < 0.01, ∗∗∗*P* < 0.001.

To further assess the therapeutic potential of targeting NLN, we conducted further validation of the effects of NR2 using a murine model. Nude mice with subcutaneously implanted xenograft tumors were randomized into two groups: one receiving a vehicle control and the other receiving NR2 at a dosage of 25 mg/kg, administered intravenously every 2–3 days. Using in vivo imaging in small animals, we observed suppression of tumor growth in mice administered NR2, with tumor sizes reduced compared to the control group (Fig. 7A and B). Consistent results were obtained through both volumetric and weight assessments (Fig. 7C and D). While the therapeutic effect of NR2 was modest in this study, statistical analyses confirmed significant intergroup differences (p < 0.05), consistent with in vitro trends. Subsequent immunohistochemical analysis of the resected tumor tissues using Ki67 and 4-HNE (a biomarker for lipid peroxidation) revealed a significant downregulation of Ki67 expression in tumors from the NR2 treatment group, indicating an inhibition of proliferation (Fig. S7A). Conversely, a pronounced increase in 4-HNE staining within the tumor tissues of the NR2 treatment group suggested heightened lipid peroxidation, implicating the onset of ferroptosis (Fig. S7B). We conducted a series of experiments to preliminarily assess the safety of NR2 in our murine model. The results demonstrated no significant changes in body weight throughout the NR2 treatment period (Fig. S7C). Hematoxylin and eosin (H&E) staining was performed on tissues from the heart, liver, kidneys, spleen, and lungs of mice in both the control (Vehicle) and NR2-treated groups. Observations indicated that NR2 treatment did not markedly alter the histological structure of these organs, with no signs of bleeding or other symptoms (Fig. S7D). Serum biochemical routine tests and hematological parameters in whole blood were also evaluated. A slight reduction in alanine transaminase (ALT) and creatinine (CREA) levels was observed; however, both remained within the normal range (Fig. S7E). The principal hematological indicators, including red blood cells (RBC), platelets (PLT), white blood cells (WBC), and lymphocytes (Lym), exhibited no significant changes, with all variations falling within normal limits (Table S3). These experimental outcomes suggest that while NR2 may induce minor deviations in certain parameters in vivo, it does not lead to severe toxic side effects, thereby confirming the preliminary safety of NR2 administration within a murine model. These outcomes substantiate that NR2 can markedly treat and retard the progression of primary lung cancer in mice, indicating that NR2 may represent a promising therapeutic target for lung cancer with clinical translational potential.

Given that primary lung cancer models provide a more accurate assessment of targeted therapeutic efficacy compared to nude mouse subcutaneous tumor models, we utilized lentivirus to overexpress the oncogene *KRAS*^G12D^ and silence the tumor suppressor gene *TP53* in adult mice to induce tumor formation. This strategy enabled the establishment of a murine model of primary lung cancer, which facilitated subsequent NR2 treatment experiments. The animals were randomly assigned to two groups: a treatment group receiving NR2 and a control group receiving the NR2 Vehicle, with wild-type (WT) mice serving as a natural control. Upon tissue harvest, macroscopic examination of the lung surface revealed a significant decrease in the number of neoplastic lesions in the treatment group (Fig. 7E). This finding was further corroborated by H&E staining of lung tissue sections, and Micro-CT imaging also demonstrated a significant reduction in the number of pulmonary lesions in the treatment group (Fig. 7F).

To evaluate NR2-induced ferroptosis in vivo, GPX4 levels were quantified in nude mouse subcutaneous tumor models and *KRAS*^G12D^/*P53*^−/−^-driven primary lung cancer mouse models, showing reduced expression after NR2 treatment (Fig. 7G and H). Rescue experiments using *KRAS*^G12D^/*P53*^−/−^ mouse lung cancer-derived organoids showed that NR2 treatment induced lipid peroxidation (BODIPY C11 staining) and suppressed proliferation (Fig. 7I; Fig. S7F). Ferrostatin-1 pretreatment reversed these effects (Fig. 7I; Fig. S7F), confirming ferroptosis-mediated cytotoxicity.

Altogether, our data support the carcinogenic potential of NLN. We elucidated the specific molecular mechanism of NLN-induced ferroptosis in lung cancer cells. Depletion of NLN inhibits m^6^A modification of *GPX4* mRNA, leading to degradation of *GPX4* mRNA, leading to ferroptosis in lung cancer cells (Fig. 7J).

## 4 Discussion

Neurolysin (NLN), a zinc metalloendopeptidase first identified in 1989, is recognized for its involvement in the degradation of neurotensin (NT) and the regulation of neurotransmitter release and signal transduction [28,29]. It has been implicated in neuroprotection following stroke, with various studies investigating its potential as a therapeutic target for enhancing brain recovery after such events [30,31]. However, its role in cancer, especially in solid tumors, remains less explored. Early studies indicated that NLN and TOP secreted from B16F10-Nex2 melanoma cells could control angiogenesis, thereby contributing to melanoma growth [32]. More recent research has highlighted the necessity of NLN for the growth of leukemic cells and progenitors, with NLN knockdown impairing the formation of respiratory chain supercomplexes (RCS), which are crucial for oxidative phosphorylation in acute myeloid leukemia(AML) [10]. Currently, there are no research reports addressing NLN in the context of lung cancer. In this study, we identify NLN may play a role in regulating ferroptosis in certain NSCLC cell models, offering new insights into mitochondrial peptidases in tumor biology. Our findings reveal that NLN is significantly upregulated in NSCLC tissues when compared to adjacent non-cancerous tissues, and this overexpression is strongly associated with poor prognosis. Furthermore, expanded profiling across NSCLC cell lines revealed substantial heterogeneity in NLN expression, indicating that its upregulation defines a distinct tumor subgroup. These results suggest that NLN may play a critical role in the development of this NLN-high subgroup of lung cancer and could represent a potential therapeutic target for these patients, thereby enhancing our understanding of NLN's function in this malignancy.

Further analysis utilizing RNA sequencing and targeted metabolomics indicated that the downregulation of NLN significantly enriched the ferroptosis pathway, thereby suggesting a strong association between NLN and ferroptosis. Characteristic assays designed to assess ferroptosis corroborated this observation. Ferroptosis is a newly identified form of regulated cell death characterized by iron-dependent phospholipid peroxidation. This process is implicated in various pathological conditions, including cancer, ischemic organ injury, and neurodegenerative diseases [33]. The induction of ferroptosis has emerged as a promising therapeutic strategy for cancer treatment [34,35]. In lung cancer, ferroptosis can be modulated through various mechanisms and signaling pathways, influencing tumor cell survival, proliferation, migration, and drug resistance. For example, certain chemotherapeutic agents, such as cisplatin, paclitaxel, and gemcitabine, can promote ferroptosis in lung cancer cells by elevating reactive oxygen species (ROS) and iron levels, as well as by inhibiting GPX4 and the Xc-system [35,36]. Additionally, natural compounds like baicalin, solasodine, and ginsenoside Rh2 can also regulate ferroptosis in lung cancer cells by targeting key factors such as Nrf2, ATF4, HSP90, and IRF1 [37,38]. Furthermore, ferroptosis can influence the immune microenvironment in lung cancer, affecting the functions of immune cells, including tumor-associated macrophages, dendritic cells, natural killer cells, and T cells [39]. Consequently, ferroptosis is crucial in lung cancer and may represent a novel target or therapeutic strategy. Currently, there is a lack of research concerning the regulation of ferroptosis by NLN, rendering this study a pioneering effort in elucidating NLN's role in the modulation of ferroptosis within the context of lung cancer. This finding is significant as it paves the way for a deeper understanding of the pathogenic mechanisms underlying lung cancer and may contribute to the development of novel therapeutic strategies.

The molecular regulatory mechanisms of ferroptosis are intricate, involving the balance of redox reactions, cysteine uptake, iron metabolism, lipid metabolism, and energy metabolism [33,40]. In our study, we employed qRT-PCR and Western blotting to examine various key genes in the ferroptosis pathway, aiming to identify the downstream factors regulated by NLN. Our findings identified GPX4 as the most critical downstream factor involved in NLN-induced ferroptosis in lung cancer cells, with its expression significantly suppressed following NLN knockdown. GPX4 is a selenoprotein that was initially discovered and purified in the 1980s by Ursini et al. [41,42]. It serves as a principal enzyme in mammalian cells, catalyzing the reduction and detoxification of peroxides. Specifically, GPX4 eliminates lipid hydroperoxides by reducing them to their corresponding alcohols or converting free H_2_O_2_ to water, a process that requires two electrons provided by glutathione (GSH) [43]. In 2014, Stockwell's laboratory identified compounds that inhibit GPX4 through metabolic profiling and demonstrated that the overexpression or knockdown of GPX4 can regulate the lethality of 12 ferroptosis inducers in cells, thereby confirming GPX4 as a key regulatory factor in ferroptosis [7]. Mice with a knockout of the GPX4 gene succumb to renal failure, and inhibitors of GPX4 have shown promise in enhancing the efficacy of treatments against drug-resistant, persistent cancer cells [44,45]. The regulation of GPX4 expression has garnered significant attention. Liu et al. found that inhibitors of the mammalian target of rapamycin (mTOR) can induce the degradation of GPX4 without affecting its gene transcription [46]. However, the mechanism by which NLN regulates GPX4 expression remains unclear.

Notably, our findings indicate that overexpression of NLN does not significantly enhance GPX4 expression at either the mRNA or protein levels, suggesting that NLN may influence GPX4 expression at the post-transcriptional level. Post-transcriptional regulation often results in mRNA degradation. Our mRNA stability experiments confirmed that the stability of *GPX4* mRNA was diminished and underwent rapid degradation following NLN knockdown. This post-transcriptional mechanism provides a compelling explanation for the weak correlation observed between basal NLN and *GPX4* mRNA levels across NSCLC cell lines in the DepMap database. Our comprehensive analysis of approximately 142 NSCLC cell lines further reveals significant heterogeneity in NLN expression itself, suggesting its regulatory impact may vary across cellular contexts. Beyond this heterogeneity, the fundamental distinction between regulatory tiers is crucial: while strong expression correlation typically arises when an upstream regulator directly governs transcriptional rates, NLN operates as a post-transcriptional stability factor that protects existing transcripts rather than initiating new transcription. In this model, the steady-state GPX4 mRNA level represents the combined output of cell-specific transcriptional activity and NLN-mediated stabilization, where the influence of NLN is masked by both its own heterogeneous expression and the dominant, highly variable transcriptional inputs across different genetic backgrounds. Thus, the weak correlation represents the expected signature of such a post-transcriptional regulatory relationship within heterogeneous cellular populations, affirming NLN's distinct function from classical transcriptional activators.

There are generally two potential mechanisms for mRNA degradation: 1) related to mRNA modification, and 2) involving the miRNA-targeted degradation system. Interestingly, our further analysis of the metabolomics data revealed that targeting NLN led to a decrease in the overall m^6^A modification level within the cell. Thus, we speculate that NLN may impact the stability of *GPX4* mRNA by regulating the m^6^A methylation modification of *GPX4*. The m^6^A modification refers to the methylation occurring on the sixth nitrogen atom of the adenine base in RNA molecules and is recognized as one of the most significant mRNA methylation modifications [47]. This modification is a reversible epigenetic alteration that dynamically regulates RNA metabolism, including splicing, transport, stability, and translation, through a variety of methyltransferases, demethylases, and RNA-binding proteins, ultimately affecting gene expression. The role of m^6^A modification in tumors is complex, as it can either promote or inhibit tumorigenesis depending on the tumor type, stage of development, and specific signaling pathways involved. Current research indicates that m^6^A modification plays a dual role in various cancers, including breast, liver, gastric, and lung cancer [48]. The relationship between m^6^A modification and mRNA stability is contingent upon both the location and the quantity of the m^6^A modification. This modification can either facilitate mRNA degradation or confer protection against it. In cases where mRNA is marked for degradation, m^6^A modification interacts with proteins such as YTH N6-methyladenosine RNA binding protein 2 (YTHDF2) and YTH domain containing 2 (YTHDC2), which facilitate the transport of mRNA from the nucleus to the cytoplasm, where it is subsequently degraded by enzymes like the CCR4-NOT deadenylase complex or the Dcp2-Dcp1a decapping complex [49]. Conversely, m^6^A modification can also serve to protect mRNA from degradation; for example, Insulin Like Growth Factor 2 mRNA Binding Protein 1 (IGF2BP) can enhance the stability of target mRNAs, such as those of the MYC gene, by promoting their m^6^A modification [50].

Through RNA dot blot experiments, we confirmed that the knockdown of NLN results in a decrease in the overall cellular m^6^A modification level. Subsequent meRIP-qPCR experiments further validated that the m^6^A modification level of *GPX4* mRNA was significantly reduced following the targeting of NLN. The dynamic m^6^A methylation modification of RNA is primarily governed by 'writers' (methyltransferases), 'erasers' (demethylases), and 'readers' (binding proteins). Methyltransferases, such as METTL3, METTL14, WTAP, and KIAA1492, form a complex that collaborates to catalyze the methylation of mRNA adenosine bases at the sixth position, known as m^6^A modification. In eukaryotes, demethylases like FTO and ALKBH5 have been identified as agents that reverse this modification [51]. By overexpressing the methyltransferase METTL3 and treating cells with the FTO inhibitor FB23-2, we were able to regulate intracellular m^6^A levels and confirm that NLN modulates the expression of GPX4 by inhibiting cellular m^6^A modification, which leads to the degradation of GPX4 mRNA and subsequently induces ferroptosis in lung cancer cells. Regarding why NLN overexpression does not increase GPX4 expression via m^6^A modification, we propose the following explanation: NLN knockdown likely reduces m^6^A modification on *GPX4* mRNA below a critical stability threshold, triggering rapid mRNA degradation. In contrast, NLN overexpression may only restore m^6^A levels to a baseline that extends mRNA half-life but does not further enhance transcriptional output or protein synthesis. This threshold-dependent regulation of m^6^A stability, combined with potential compensatory mechanisms (e.g., feedback inhibition or demethylation), may explain the lack of additive effects from NLN overexpression. But the precise mechanism by which NLN regulates the m^6^A methylation level of GPX4 remains to be elucidated. Targeting NLN has been shown to decrease the m^6^A modification level of *GPX4* mRNA, leading to its instability. Therefore, we hypothesize that the m^6^A modification site on *GPX4* mRNA is not situated within the poly(A) tail but rather within the internal region of the mRNA. It is possible that NLN modulates the binding and release of RNA-binding proteins or protein complexes, such as IGF2BP, to GPX4 mRNA via m^6^A modification, thus influencing its stability. This study is the first to demonstrate that the inhibition of NLN induces ferroptosis in lung cancer cells, elucidating the regulation of *GPX4* expression by m^6^A modification and enriching the complexity of the ferroptosis regulatory network. It also presents a novel approach and potential for inducing tumor ferroptosis.

After elucidating NLN as a potential therapeutic target and clarifying the molecular mechanisms by which inhibiting this target induces ferroptosis in lung cancer cells, we aim to further explore the clinical value of the NLN target, which could provide a scientific basis for its clinical translation. Although numerous reports exist on specific inhibitors of NLN, few have been utilized for subsequent research to develop clinical value, and there are currently no commercial inhibitors available [52]. Therefore, based on existing literature, we synthesized the specific inhibitor 3-[(2S)-1-[(3R)-3-(2-Chlorophenyl)-2-(2-fluorophenyl) pyrazolidin-1-yl]-1-oxopropa n-2-yl]-1-(adamantan-2-yl)urea, referred to as NR2 for ease of description, which has been reported to specifically inhibit the function of NLN in research on acute myeloid leukemia (AML) [10,26]. Through in vitro IC_50_ testing on various lung cancer cell lines and cytotoxicity experiments on lung cancer organoids, NR2 has been confirmed to induce tumor cell death and exhibit significant anti-tumor effects. Further evaluation of NR2's efficacy and safety in vivo, using subcutaneous tumor models and primary lung cancer mouse models, demonstrated that NR2 significantly inhibited the growth of subcutaneous tumors in mice and markedly suppressed the progression of primary lung cancer. This indicates that NR2 has anti-tumor effects in vivo. Additionally, C11 staining, GPX4 expression detection, and tissue lipid peroxidation staining confirmed that NR2 can induce ferroptosis in lung cancer cells. This suggests that NLN, as a potential therapeutic target for non-small cell lung cancer, may enhance efficacy or address drug resistance issues by inducing ferroptosis in lung cancer. Future research should prioritize pharmacokinetic experiments in murine models to enhance the translational potential of NR2. Furthermore, in vitro biochemical assays should be conducted to evaluate the binding affinity of NR2 to NLN, thereby confirming the specificity of this interaction. Additionally, it is essential to initiate combination therapy studies with conventional targeted treatments for lung cancer, as this may yield novel strategies for managing this disease.

## 5 Conclusion

In conclusion, to address the limited number of patients benefiting from existing targets in the targeted therapy of NSCLC and the challenges posed by drug resistance, our study focused on screening and investigating new targets through high-throughput proteomics. We demonstrated that NLN could serve as a novel potential therapeutic target for NSCLC. Our findings confirmed that NLN exhibits oncogenic properties; targeted inhibition of NLN was shown to induce ferroptosis in lung cancer cells. In vivo experiments further indicated that NLN inhibition suppressed the growth of subcutaneous tumors in mice. Additionally, we elucidated the specific molecular mechanism by which NLN induces ferroptosis in lung cancer cells: the knockdown of NLN inhibits the m^6^A modification of GPX4 mRNA, leading to the degradation of GPX4 mRNA and subsequently triggering ferroptosis in lung cancer cells. To further validate the clinical relevance of NLN, we synthesized a small molecule inhibitor specific to NLN, named NR2. Through both in vitro and in vivo experiments, we demonstrated that NR2 can induce tumor cell death and exhibits significant anti-tumor effects, thereby providing a research foundation for the clinical application of NLN as a therapeutic target.

## Ethical approval

This study was approved by the Ethics Committee on Biomedical Research, West China Hospital of Sichuan University (Chengdu China).

## Funding

This work was supported by grants from the 10.13039/501100001809National Natural Science Foundation of China (82472703, 82173251, 82470109, 92159302), the Noncommunicable Chronic Diseases-National Science and Technology Major Project of China (2024ZD0529500/2024ZD0529504), the 1.3.5 Project for Disciplines of Excellence, 10.13039/501100013365West China Hospital, Sichuan University (ZYYC23027), and the 1.3.5 Project of State Key Laboratory of Respiratory 10.13039/100018696Health and Multimorbidity, 10.13039/501100013365West China Hospital, Sichuan University (RHM24208).

## CRediT authorship contribution statement

**Lei Liu:** Conceptualization, Data curation, Formal analysis, Investigation, Methodology, Validation, Writing – original draft, Writing – review & editing. **Yinyun Ni:** Data curation, Formal analysis, Investigation, Methodology, Validation, Writing – original draft, Writing – review & editing. **Ying Yang:** Data curation, Formal analysis, Investigation, Methodology, Writing – review & editing. **Gan Zhang:** Investigation, Methodology. **Shengqiang Mao:** Data curation, Software. **Ningning Chao:** Data curation, Investigation. **Menglin Yao:** Data curation, Investigation. **Chengdi Wang:** Conceptualization, Funding acquisition, Supervision, Writing – original draft, Writing – review & editing. **Li Zhang:** Conceptualization, Funding acquisition, Supervision, Writing – original draft, Writing – review & editing.

## Declaration of competing interest

The authors declare that they have no known competing financial interests or personal relationships that could have appeared to influence the work reported in this paper.

## Data Availability

Data will be made available on request.
