# Supplementary material for: Loss of NLN suppresses lung cancer progression by inducing ferroptosis through downregulating m6A methylation of GPX4
Source: Redox Biol. 2025 Nov 11;88:103928. doi: 10.1016/j.redox.2025.103928 (PMC12664413; doi:10.1016/j.redox.2025.103928)
Supplement: Multimedia component 1 [file mmc1.docx]

**Supplemental information**

**Supplemental Figures**

**
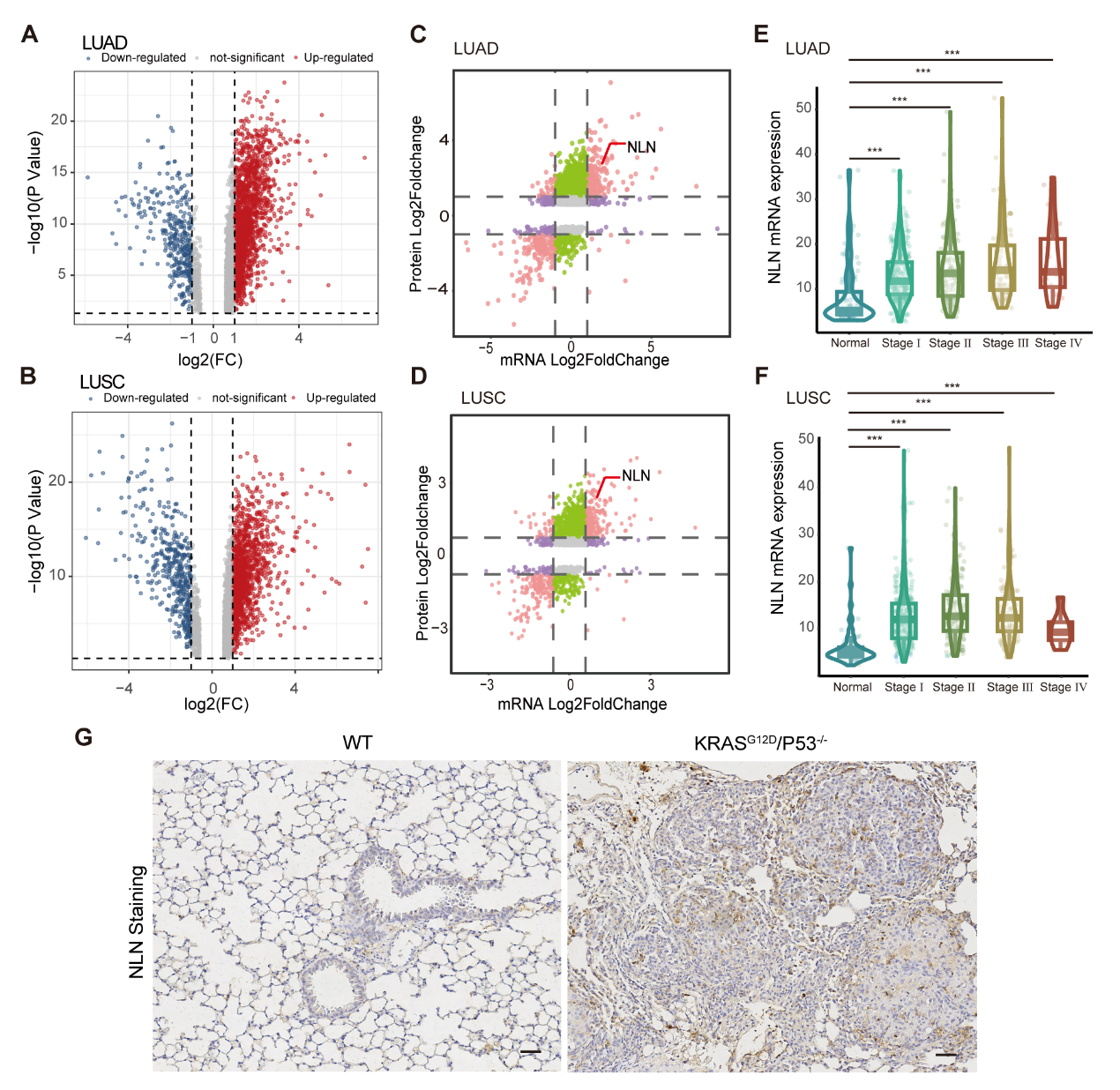
**

**Figure S1. NLN is highly expressed in the early stage of NSCLC and in mouse primary lung cancer tissues**

1. Volcano plot of differential protein analysis in LUAD. The dashed line indicates the threshold for significant differential expression. (B) Volcano plot of differential protein analysis in LUSC. (C, D) Integrated proteomic and transcriptomic analysis of NLN expression in LUAD and LUSC tumor tissues versus NAT. (E) TCGA data analysis showing NLN mRNA expression across different stages of LUAD. (F) TCGA data analysis showing NLN mRNA expression across different stages of LUSC. Each dot represents an individual sample, and the bars indicate median expression levels. (G) Expression of NLN in tumor tissues of primary lung adenocarcinoma mouse models. WT: wild-type mice; KRAS^G12D/P53-/-^: mice overexpressing KRAS^G12D^ with TP53 gene deletion. Scale bar: 50μm.(left) and 200μm (right). ****P* < 0.001.


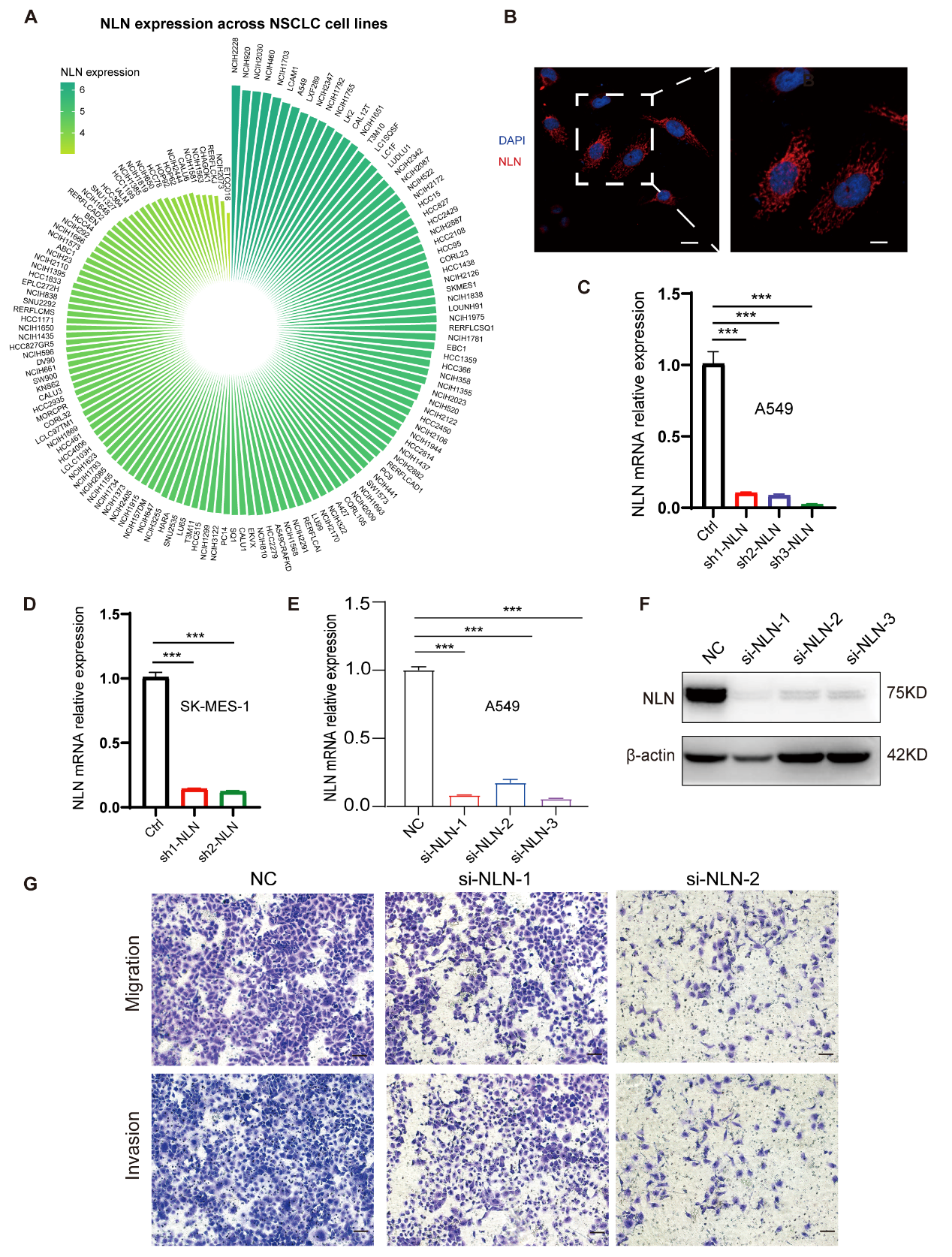


**Figure S2. SiRNA knockdown of NLN inhibits lung cancer cell migration and invasion**

(A) NLN mRNA expression across the full panel of ~142 NSCLC cell lines available in the DepMap database (Public 25Q3). (B) Immunofluorescence staining of NLN in A549 cells. Blue: nucleus (DAPI); Red: NLN. Scale bars: 50μm (left) and 30μm (right). (C) qRT-PCR detection of knockdown efficiency of three sh-NLN sequences in A549 cells. (D) qRT-PCR detection of knockdown efficiency of two sh-NLN sequences in SK-MES-1 cells. (E) qRT-PCR detection of knockdown efficiency of si-NLN sequences in A549 cells. (F) Western blot analysis of knockdown efficiency of si-NLN sequences. (G) Migration and invasion assay results of A549 cells in the control group (NC) and NLN knockdown group (si-NLN). Scale bar: 100μm. Error bars represent standard error of the mean (SEM). **P* < 0.05, ***P* < 0.01, ****P* < 0.001, *****P* < 0.0001.


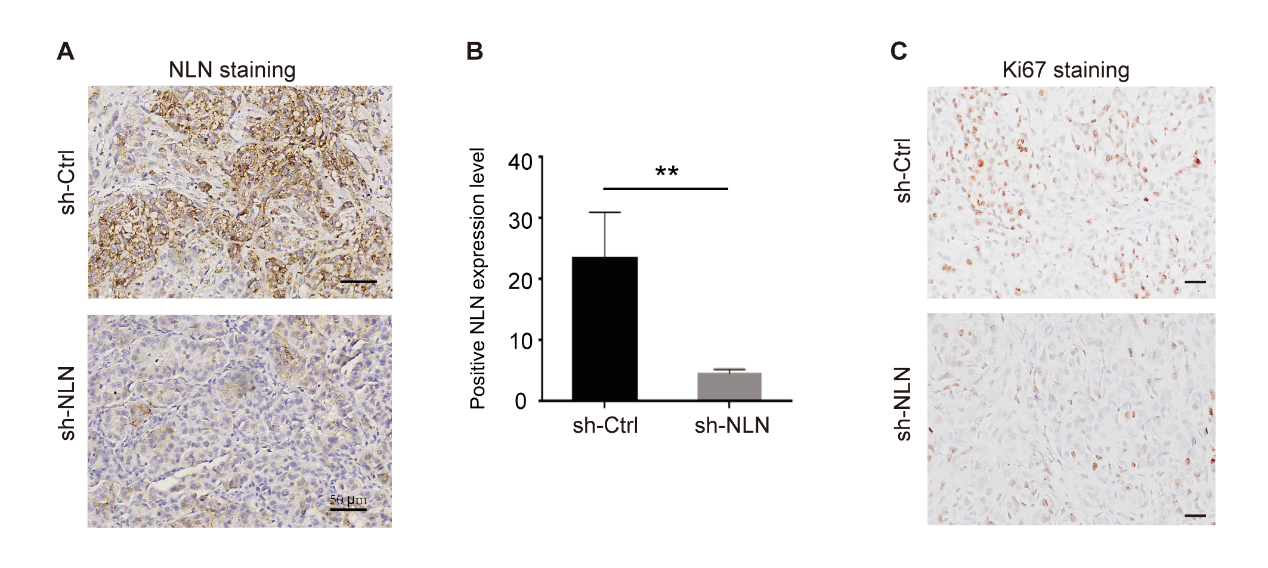


**Figure S3. Molecular assessment of tumor proliferation**

1. Immunohistochemical staining of NLN in subcutaneous tumor tissues from control (sh-Ctrl) and NLN knockdown (sh-NLN) groups. (B) Graphical results showing the NLN levels in (A). (C) Immunohistochemical staining of Ki67 in subcutaneous tumor tissues from control (sh-Ctrl) and NLN knockdown (sh-NLN) groups. Scale bars: 500μm (left) and 100μm (right).

**
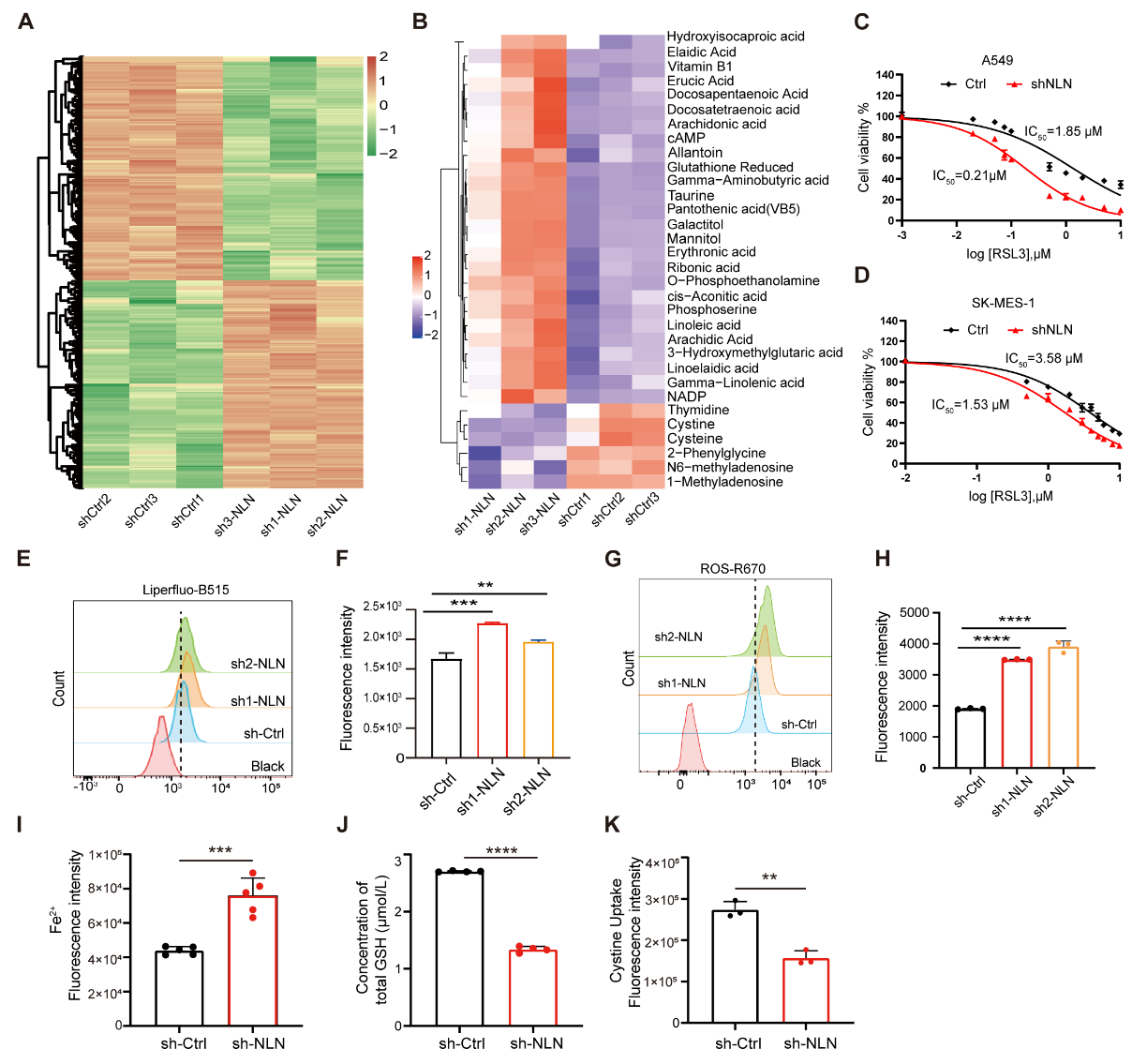
**

**Figure S4.** **Multiple ferroptosis characterization detection**

1. Clustering analysis of differentially expressed transcripts from Nanopore sequencing following stable knockdown of NLN. (B) Hierarchical clustering heatmap of significantly different metabolites following NLN knockdown. (C, D) Dose-response curves of A549 (C) and SK-MES-1 (D) cells with or without NLN knockdown treated with RSL3. The leftward shift and decreased IC50 value indicate enhanced sensitivity to ferroptosis. (E) Flow cytometric analysis of Liperfluo staining. (F) Mean fluorescence intensity plots of Liperfluo show the lipid peroxidation on the cell membrane. (G) Flow cytometric analysis of CellROX™ Deep Red staining in A549 cells, indicating increased ROS levels. (H) Statistical plot of average fluorescence intensity in (G). (I) FerroOrange staining for ferrous ion detection in A549 cells, showing increased intracellular ferrous iron levels. (J) Enzymatic assay for total glutathione (GSH) levels, indicating a decrease post-NLN knockdown. (K) Cystine uptake assay, indirectly reflecting xCT system activity, showing a reduction following NLN knockdown. **P* < 0.05, ***P* < 0.01, ****P* < 0.001, *****P* <0.0001.

**
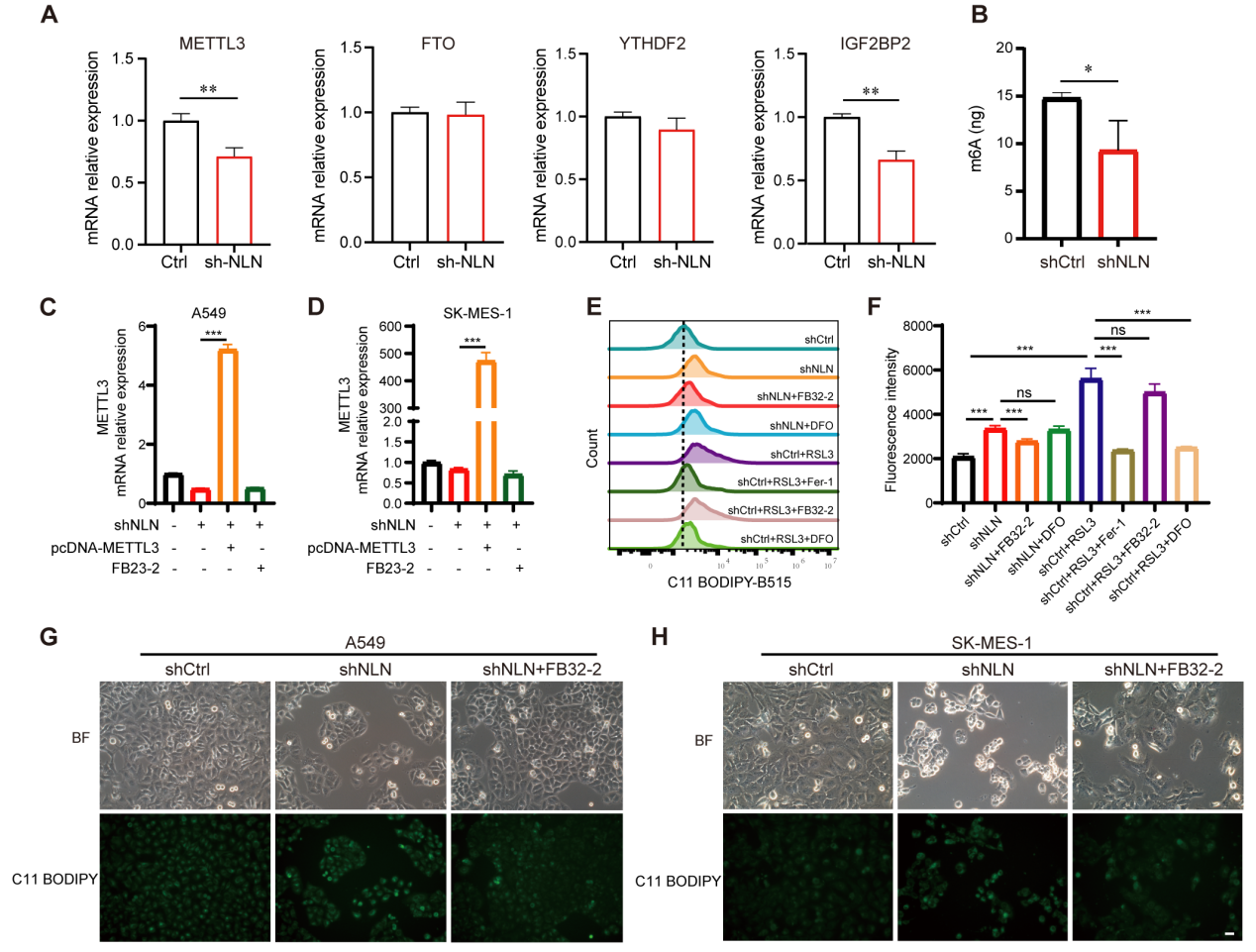
**

**Figure S5. Additional analyses of m6A modification and lipid peroxidation**

1. qRT-PCR to detect changes in the expression of key enzyme mRNA levels during m6A modification. (B) EpiQuik™m6A RNA Methylation Quantification Kit quantified the changes in m6A modification levels after knockdown of NLN in A549 cells. (C) qRT-PCR analysis of METTL3 mRNA levels in A549 cells overexpressing METTL3 or treated with FB23-2. (D) qRT-PCR analysis of METTL3 mRNA levels in SK-MES-1 cells overexpressing METTL3 or treated with FB23-2. (E) Representative flow cytometry plots showing BODIPY C11 fluorescence intensity in A549 control and NLN-knockdown cells under different treatment conditions. (F) Statistical quantification of average BODIPY C11 fluorescence intensity from (E). (G) Fluorescence microscopy photographed images of C11-stained A549 cells after different treatment groups. BF: bright field, C11 BODIPY: lipid peroxidation indicator staining, green fluorescence. (H) Fluorescence microscopy photographed images of C11-stained SK-MES-1 cells after different treatment groups. Scale Bar:100μm. Error bars represent standard error of the mean (SEM). *P < 0.05, **P < 0.01, ***P < 0.001.

**
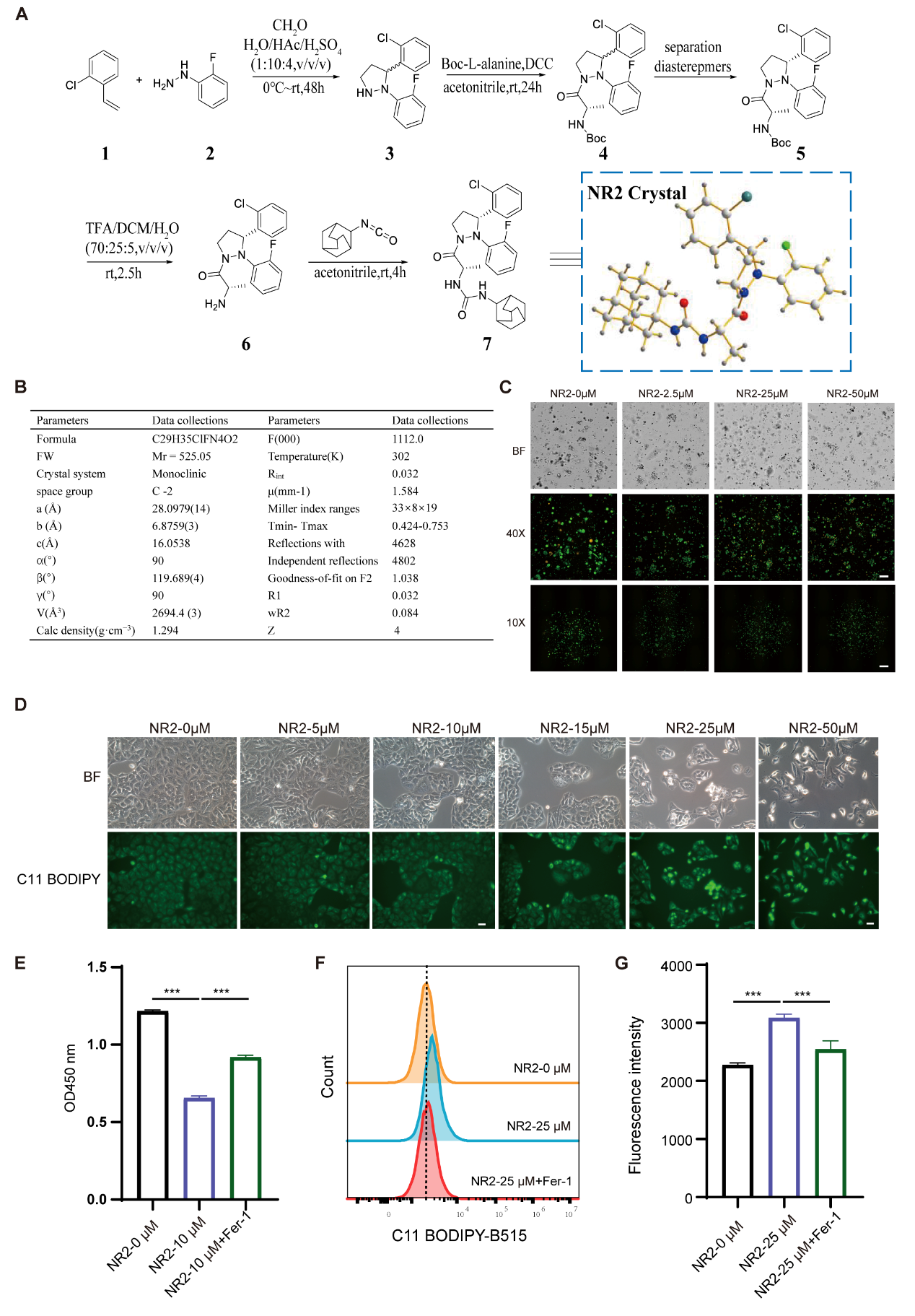
**

**Figure S6. characterization of NR2.**

(A) Synthesis route of the NR2 small molecule compound. (B) Relevant crystal parameters of NR2. (C) High-content imaging of lung adenocarcinoma cell carcinoma organoids treated with NR2. (D) Fluorescence microscopy images of A549 cells treated with NR2, demonstrating increased green fluorescence due to lipid peroxidation. (E). The CCK-8 assay indicated the cell viability of A549 cells treated with NR2 and Fer-1. (F). Representative flow cytometry plots showing BODIPY C11 fluorescence intensity in A549 cells treated with NR2 and Fer-1. (G) Statistical
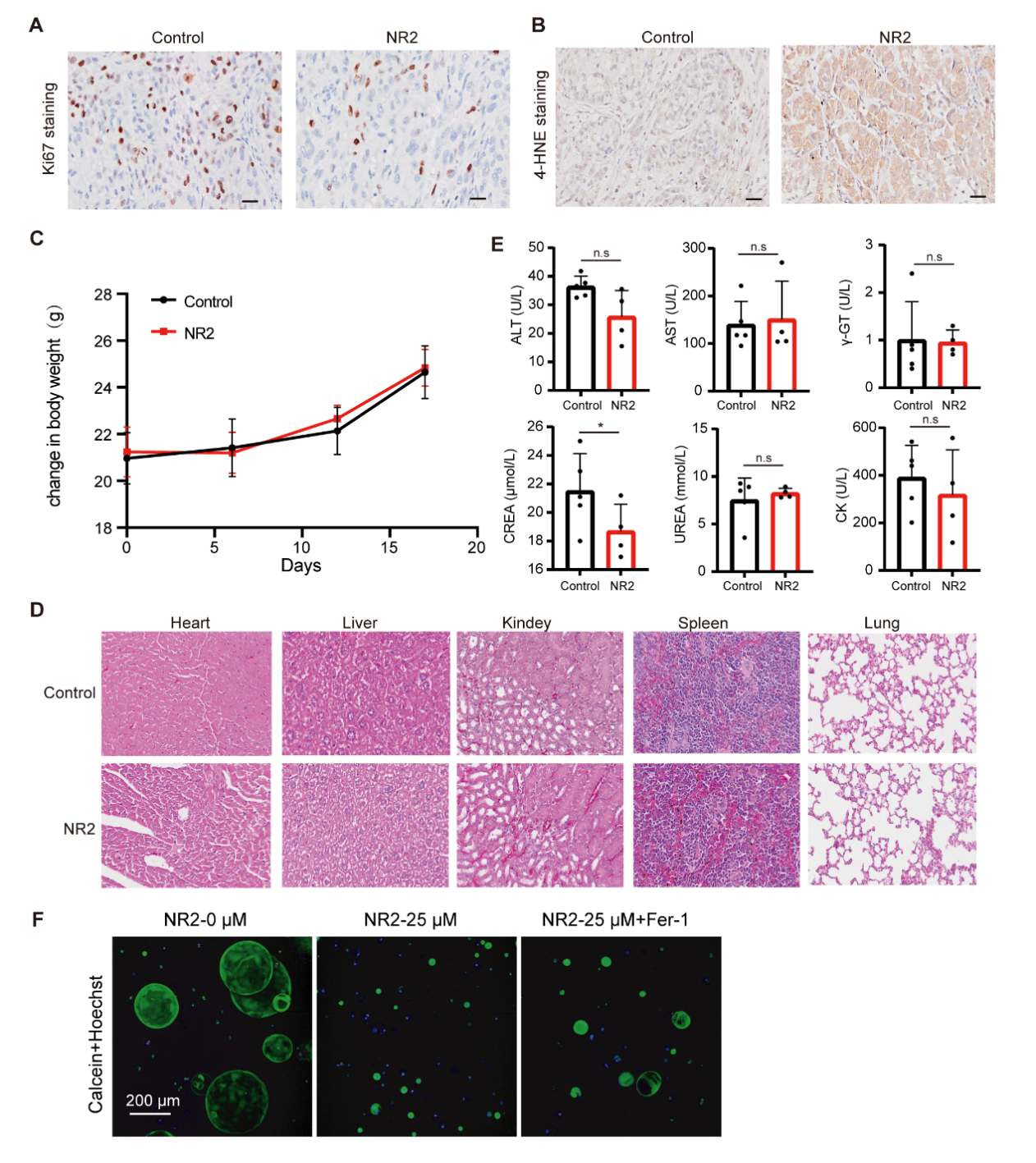
quantification of average BODIPY C11 fluorescence intensity from (F).

**Figure S7: Safety assessment of NR2 in mice**

(A) Ki67 immunohistochemical staining of subcutaneous tumor tissue. Scale bars: 50μm. (B) Immunohistochemical staining for 4-HNE (subcutaneous tumor tissue). Scale bars: 50μm. (C) Body weight monitoring of mice throughout the NR2 treatment period, showing no significant changes. (D) HE staining of tissues from major organs showing no signs of bleeding or other symptoms, suggesting preliminary safety of NR2 administration.(E) Serum biochemical routine tests and hematological parameters, indicating slight reductions in alanine transaminase (ALT) and creatinine (CREA) levels, but within normal ranges. (F) High-content confocal microscopy imaging of KRAS^G12D^/P53^−/−^ mouse lung cancer-derived organoids treated with NR2 and Fer-1. Green fluorescence indicates live cells stained with calcein; blue fluorescence corresponds to cell nuclei stained with Hoechst. Error bars represent standard error of the mean (SEM). **P* < 0.05.

**Supplemental Tables**

**Table S1. Primary antibodies used in this study**

| **Antibody for WB** | **Host** | **Company** | **Cat#** | **Dilution** |
| --- | --- | --- | --- | --- |
| NLN antibody( OTI1D6) | Rabbit | OriGene | TA504178 | 1:1000 |
| NLN antibody | Mouse | SANTA CRUZ | sc-398613 | 1:500 |
| ACSL4 antibody | Rabbit | CST | 38493T | 1:1000 |
| GPX4 antibody | Rabbit | CST | 52455 | 1:1000 |
| Keap1 antibody | Rabbit | CST | 8047 | 1:1000 |
| SLC7A11 antibody | Rabbit | ABclonal | A2413 | 1:1000 |
| HMGCR antibody | Rabbit | ABclonal | A16875 | 1:1000 |
| FTH1 antibody | Rabbit | CST | 4393 | 1:1000 |
| FASN antibody | Rabbit | CST | 3180T | 1:1000 |
| GAPDH antibody | Mouse | Proteintech | 60004-1-Ig | 1:10000 |
| beta-Tublin antibody | Rabbit | Abcam | ab6046 | 1:5000 |
| beta-Actin antibody | Mouse | Proteintech | 66009-1-Ig | 1:4000 |
| m6A antibody | Rabbit | SYSY Antibodies | 202003 | 1:1000 |
| m⁶A Monoclonal Antibody | Mouse | Proteintech | 68055-1-Ig | 1：2000 |
|  |  |  |  |  |
| **Antibody for IHC** |  |  |  |  |
| 4HNE | Rabbit | Abcam | ab46545 | 1:100 |
| Ki-67 | Rabbit | CST | 9027 | 1:400 |
| NLN | Rabbit | 14763-1-AP | 9664 | 1:200 |

**Table S2. Primer sequences for PCR used in this study**

| **Genes** | **Primer sequences** |
| --- | --- |
| NLN- Forward (human)-For | 5’ TGCTCGGTATTGAGGAAGTAACT 3’ |
| NLN- Reverse (human)-Rev | 5’ TGGGGAAAGTCTAGCATGGT 3’ |
| GPX4- Forward (human)-For | 5’ GAGGCAAGACCGAAGTAAACTAC3’ |
| GPX4- Reverse (human)-Rev | 5’ CCGAACTGGTTACACGGGAA 3’ |
| PTGS2 (human)-For | 5’ CGGTGAAACTCTGGCTAGACAG 3’ |
| PTGS2 (human)-Rev | 5’ GCAAACCGTAGATGCTCAGGGA 3’ |
| ACSL4 (human)-For | 5’ GCTATCTCCTCAGACACACCGA 3’ |
| ACSL4 (human)-Rev | 5’ AGGTGCTCCAACTCTGCCAGTA 3’ |
| SLC7A11 (human) -For | 5’ TCCTGCTTTGGCTCCATGAACG3’ |
| SLC7A11 (human)- Rev | 5’ AGAGGAGTGTGCTTGCGGACAT 3’ |
| FTH1 (human) -For | 5’ TGAAGCTGCAGAACCAACGAGG 3’ |
| FTH1 (human)- Rev | 5’ GCACACTCCATTGCATTCAGCC 3’ |
| FTL (human) -For | 5’ TACGAGCGTCTCCTGAAGATGC 3’ |
| FTL (human)- Rev | 5’ GGTTCAGCTTTTTCTCCAGGGC 3’ |
| NQO1 (human) -For | 5’ AGAAAGGATGGGAGGTGGTG3’ |
| NQO1(human)- Rev | 5’ ATATCACAAGGTCTGCGGCT3’ |
| METTL3 (human) -For | 5’ CTATCTCCTGGCACTCGCAAGA 3’ |
| METTL3 (human)- Rev | 5’ GCTTGAACCGTGCAACCACATC 3’ |
| FTO (human) -For | 5’ CCAGAACCTGAGGAGAGAATGG 3’ |
| FTO (human)- Rev | 5’ CGATGTCTGTGAGGTCAAACGG 3’ |
| YTHDF2 (human) -For | 5’ TAGCCAGCTACAAGCACACCAC 3’ |
| YTHDF2 (human)- Rev | 5’ CAACCGTTGCTGCAGTCTGTGT 3’ |
| IGF2BP2 (human) -For | 5’ GTTGGTGCCATCATCGGAAAGG3’ |
| IGF2BP2 (human)- Rev | 5’ TGGATGGTGACAGGCTTCTCTG 3’ |
| MeRIP-position#1- For | CTCCATGCACGAGTTTTCC |
| MeRIP-position#1- Rev | TTTACTTCGGTCTTGCCTCA |
| MeRIP-position#2- For | GGAGCCCCTGGTGATAG |
| MeRIP-position#2- Rev | CATGAGTGCCGGTGGAA |
| MeRIP-position#3- For | CAGACCCGAAAATCCAGC |
| MeRIP-position#3- Rev | TCTGTTTATTCCCACAAGGT |
| 18S (human) -For | 5’ GTAACCCGTTGAACCCCATT 3’ |
| 18S (human)- Rev | 5’ CCATCCAATCGGTAGTAGCG 3’ |

**Table S3: Routine blood test before and after NR2 administration.**

| Detection indicators | No.1 | No.2 | No.3 | No.4 | No.5 | No.6 | No.7 | No.8 | No.9 | reference range |
| --- | --- | --- | --- | --- | --- | --- | --- | --- | --- | --- |
| WBC (10^9^/L) | 1.2 | 1.5 | 1.1 | 1.5 | 0.9 | 0.9 | 1.1 | 0.9 | 1.2 | 0.8-6.8 |
| Lym# (10^9^/L) | 0.9 | 1.3 | 0.8 | 1.2 | 0.7 | 0.7 | 0.8 | 0.7 | 0.9 | 0.7-5.7 |
| Mon# (10^9^/L) | 0.1 | 0.0 | 0.1 | 0.1 | 0.0 | 0.0 | 0.1 | 0.0 | 0.1 | 0.0-0.3 |
| Gran# (10^9^/L) | 0.2 | 0.2 | 0.2 | 0.2 | 0.2 | 0.2 | 0.2 | 0.2 | 0.2 | 0.1-1.8 |
| Lym% (%) | 72.9 | 85.9 | 75.8 | 81.8 | 72.6 | 76.3 | 72.2 | 73.0 | 70.8 | 55.8-90.6 |
| Mon% (%) | 9.2 | 3.4 | 8.3 | 4.7 | 9.0 | 6.9 | 8.4 | 9.0 | 9.1 | 1.8-6.0 |
| Gran% (%) | 17.9 | 10.7 | 15.9 | 13.5 | 18.4 | 16.8 | 19.4 | 18.0 | 20.1 | 8.6-38.9 |
| RBC (10^12^/L) | 7.67 | 9.09 | 9.05 | 9.00 | 8.77 | 8.54 | 8.15 | 7.69 | 8.32 | 6.36-9.42 |
| HGB (g/L) | 120 | 140 | 143 | 136 | 137 | 132 | 134 | 123 | 132 | 110-143 |
| HCT (%) | 37.9 | 44.7 | 45.5 | 44.1 | 43.5 | 42.8 | 42.5 | 39.6 | 42.0 | 34.6-44.6 |
| MCV (fL) | 49.5 | 49.2 | 50.3 | 49.1 | 49.7 | 50.2 | 52.2 | 51.5 | 50.5 | 48.2-58.3 |
| MCH (pg) | 15.6 | 15.4 | 15.8 | 15.1 | 15.6 | 15.4 | 16.4 | 15.9 | 15.8 | 15.8-19.0 |
| MCHC (g/L) | 316 | 313 | 314 | 308 | 314 | 308 | 315 | 310 | 314 | 302-353 |
| RDW (%) | 11.6 | 12.2 | 12.4 | 12.6 | 12.0 | 12.7 | 12.8 | 12.5 | 11.7 | 13.0-17.0 |
| PLT (10^9^/L) | 764 | 434 | 626 | 659 | 890 | 645 | 567 | 635 | 600 | 450-1590 |
| MPV (fL) | 4.3 | 4.4 | 4.5 | 4.4 | 4.3 | 4.3 | 4.5 | 4.3 | 4.4 | 3.8-6.0 |
| PDW (fL) | 16.3 | 16.8 | 16.9 | 16.2 | 16.4 | 16.2 | 17.2 | 16.1 | 16.4 | 10-18 |
| PCT (%) | 0.328 | 0.190 | 0.281 | 0.289 | 0.382 | 0.277 | 0.255 | 0.273 | 0.264 | 0.108-0.282 |

WBC: White Blood Cell Count, Neu: Neutrophils, Lym: Lymphocytes, Mon: Monocytes, Eos: Eosinophils, Bas: Basophils, RBC: Red Blood Cell Count, HGB: Hemoglobin, HCT: Hematocrit, MCV: Mean Corpuscular Volume, MCH: Mean Corpuscular, Hemoglobin, MCHC: Mean Corpuscular Hemoglobin Concentration, RDW-CV: Red Cell Distribution Width - Coefficient of Variation, RDW-SD: Red Cell Distribution Width - Standard Deviation, PLT: Platelet Count, MPV: Mean Platelet Volume, PDW: Platelet Distribution Width, PCT: Platelet Crit0, No.1 to No.5 are the solvent control group (n=5 mice), No.6 to No.9 are the NR2 treatment group (n=4 mice).
